# Attentional selection of levels within hierarchically organized figures is mediated by object-files

**DOI:** 10.3389/fnint.2014.00091

**Published:** 2014-12-16

**Authors:** Mitchell J. Valdés-Sosa, Jorge Iglesias-Fuster, Rosario Torres

**Affiliations:** Cognitive Neuroscience, Cuban Center for NeuroscienceHavana, Cuba

**Keywords:** attention, object-files, hierarchical perception, global, local, attentional blink, spatial frequencies

## Abstract

Objects frequently have a hierarchical organization (tree-branch-leaf). How do we select the level to be attended? This has been explored with compound letters: a global letter built from local letters. One explanation, backed by much empirical support, is that attentional competition is biased toward certain spatial frequency (SF) bands across all locations and objects (a SF filter). This view assumes that the global and local letters are carried respectively by low and high SF bands, and that the bias can persist over time. Here we advocate a complementary view in which perception of hierarchical level is determined by how we represent letters in object-files. Although many properties bound to an object-file (i.e., position, color, even shape) can mutate without affecting its persistence over time, we posit that same object-file cannot be used to store information from different hierarchical levels. Thus, selection of level would be independent from locations but not from the way objects are represented at each moment. These views were contrasted via an attentional blink paradigm that presented letters within compound figures, but only one level at a time. Attending to two letters in rapid succession was easier if they were at the same-compared to different-levels, as predicted by both accounts. However, only the object-file account was able to explain why it was easier to report two targets on the same moving object compared to the same targets on distinct objects. The interference of different masks on target recognition was also easier to predict by the object-file account than by an SF filter. The methods introduced here allowed us to investigate attention to hierarchical levels and to object-files within the same empirical framework. The data suggests that SF information is used to structure the internal organization of object representations, a process understood best by integrating object-file theory with previous models of hierarchical perception.

## Introduction

### Object based attention

Although we can choose to attend to anything that happens at a given spatial location (i.e., the goal zone in a match of the FIFA World Cup), or to a specific feature (i.e., find the black uniforms in the playfield), we often focus on visual objects. The last alternative is especially sensible from an ecological point of view, given that most of our interactions with the world are precisely directed at objects (i.e., we grasp/eat/avoid/or-flee-from objects or we boo at them if they fail to score a goal). These alternatives for defining the units of selection are known respectively as spatial-based, feature-based and object-based attention (Serences et al., 2004).

The two-target test (TTT) was developed to identify which of these units are used in attention in a specific scenario. This test assumes that there should be little competition between two pieces of information arising within the same unit of attentional selection, in contrast to strong competition when these pieces originate from distinct units (Duncan, 1984; Kravitz and Behrmann, 2011). Thus, accuracy of reports about two targets have been compared when they belong to the same location/feature/object and when they do not. Note that TTT elegantly keeps several confounding factors other than attention (such as the number of perceptual decisions, working memory load, and response competition) constant across the focused/divided attention comparison.

Use of TTT has shown that under many circumstances object-based attention overrides spatial-based and feature-based mechanisms. For instance, it is easier to discriminate two features belonging to a single object than if they are split between two objects, even if these are spatially superimposed (Neisser and Becklen, 1975; Duncan, 1984). Most of the early work on object-based attention used stationary visual objects (with invariant features) as stimuli that were presented with abrupt onsets and offsets. However, in real life, objects move around and mutate in their proprieties (i.e., soccer players run around the field and can collapse).

In order to conceptualize these dynamic traits, Kahneman et al. (1992), proposed the concept of object-files as mid-level visual representations that would bind the present state of an object to its preceding history, thus “integrating visual information across time to represent a unitary object moving or changing within an ongoing perceptual experience” (Treisman, 1992). These temporary representations also would bind together different features. Only a limited number of object-files can be handled by attention at the same time (Scholl, 2007), consistent with the biased competition model (Desimone and Duncan, 1995), in which attentional competition implies mutual inhibition of object representations.

Changes in a scene are interpreted as updates to an existing object-file if they do not violate spatio/temporal predictability (plausible trajectories) or if the magnitude of feature changes is small (Scholl, 2007). Object-files can survive brief occlusions by other objects or interference from visual masks (Scholl, 2007; Wutz and Melcher, 2013). However, other changes cannot be assimilated into existing object-files and consequently trigger the creation of a new representation (Mitroff et al., 2004, 2005a,b; Scholl, 2007). Updating an attended file is thought to be less attentional demanding than creating a new object-file (Moore et al., 2007), or shifting attention from one file to another. We will describe a modification of the TTT that optimizes it for research on dynamically changing object-files.

### Attention to hierarchical levels

**Figure 1:**
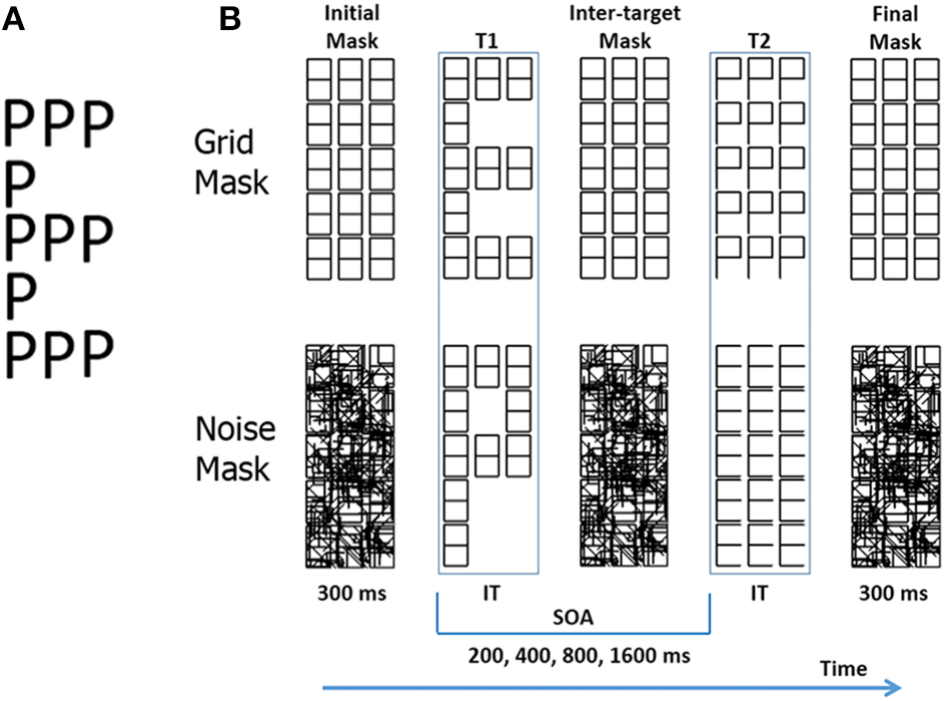
**(A)** A compound letter (a.k.a. Navon pattern). **(B)** Trial structure represented with schematic stimuli in the Experiments 1 and 2, with a grid mask (above), and with a noise mask (below). The example is one of the four types of trials: a global/local transition. IT: individually titrated durations. See **Figure 2** for more realistic gray tone depictions.

Objects frequently possess different hierarchical levels, spanning from the entire entity to increasingly finer subdivisions (e.g., tree-branch-leaf). This has been studied in the laboratory with compound letters: a global letter made out of local letters (see Figure 1A). In what is known as the Navon (1977) task, subjects are asked to make speeded letter identifications from compound figures. The letters are easily recognized when attention focused on only one echelon (usually faster for the global case), but with more difficulty when attention is divided between the two levels (Navon, 1977; Kimchi, 2014). In the latter competition it is the global level that habitually dominates.

Is there any relationship between object-based attention and attention to hierarchical levels? Unfortunately, these two research topics have been largely studied in isolation from each other (but see Vecera et al., 2000). Some work has equated selection of hierarchical levels with spatial-based attention (Stoffer, 1993; Kim et al., 1999). The idea is that global letters would require larger attentional windows than the smaller local letters. Another proposal equates selection of hierarchical levels with the selection of spatial frequency bands, with global and local information respectively mediated by relatively lower and higher frequencies (Shulman et al., 1986; Shulman and Wilson, 1987; Robertson, 1996; Flevaris et al., 2011a). We will later examine this idea of spatial frequency (SF) filters in more detail. To be fair, proponents of this hypothesis recognize that other cues can be used to signal hierarchical level (e.g., Flevaris et al., 2014).

To our knowledge, the object-file concept has not been used to understand hierarchical perception. Regrettably TTT, the mainstay of object-based attention research, cannot be fully deployed with traditional compound letters. Although traditional Navon figures admit two targets for divided attention, only one is available for focused attention. This precludes the elegant controls inherent to TTT. To overcome this limitation, compound letters and the TTT must be modified.

### A common empirical framework

To pull together research on object-files and hierarchical perception we must use a variant of the TTT that allows use of moving mutable objects, and also of compound letters. We have called this method the two-sequential-target test (TSTT). The TSTT essentially spreads the two targets of TTT over time (see Valdes Sosa et al., 2003) with variable stimulus onset asynchronies (SOAs). This allows use of the attentional blink (see reviews by Egeth and Yantis, 1997; Arnell et al., 2006; Dux and Marois, 2009). The attentional blink is an interference to the recognition of one visual target (T2) induced by the previous identification of another target (T1). This occurs typically when the targets are separated in time by less than 500 ms, and their availability has been curtailed by visual masks. The attentional blink is eliminated when T1 is ignored, which allows attention to focus on T2 (Raymond et al., 1992; Duncan et al., 1994).

Placing the targets in TSTT astride a spatial, a feature, or an object boundary, allows us to identify the units of attention in a given situation. If an attentional blink is increased by crossing a boundary (compared to avoiding it), then that boundary probably defines a legitimate unit of attentional selection. One instantiation of TSTT uses two rotating random dot kinetograms that are perceived as superimposed (but transparent) visual surfaces sliding over each other (Valdes-Sosa et al., 2000; Pinilla et al., 2001). Observers are asked to report two sequential jerks in motion direction (T1 and T2). The reports are always accurate for T1. They are also accurate for T2 if it affects the same-surface as T1, but a large attentional blink is produced if T2 switches surfaces (unless T1 is ignored).

Similar results have been reported by other groups (Mitchell et al., 2003, 2004; Reynolds et al., 2003; Khoe et al., 2005, 2008; Ciaramitaro et al., 2011). The findings can be understood if we assume that each surface creates an object-file. Updating the file for an already attended object-file is easier than shifting attention to another one. The TSTT has also been used with serial presentation of images of objects which can mutate their properties (Raymond, 2003; Valdes Sosa et al., 2003; Kellie and Shapiro, 2004). The attentional blink is ameliorated when the two targets are construed as variations of the same object-file, but will be large when they are interpreted as distributed between competing object-files, or when T2 causes the creation of a new object-file.

The TSTT can also be used with compound letters (Lopez et al., 2002), if one can uncouple the presentation of global and local letters over time. We achieve this by *level specific letter presentation*. This method presents a grid of 15 “8” symbols (Figure 1B). For brief periods of time, either some segments within the “8” symbols disappear (unmasking local letters), or complete “8” symbols disappear within the grid (unmasking a global letter). Thus, at any instance letters are shown at only one level, while easily detectable (and ignorable) patterns are presented at the other. This represents an important difference with traditional Navon figures, in which letters are always simultaneously present at both levels. The original grid, or a noise pattern, can be used to limit the persistence of the target letters, a requirement for the attentional blink as mentioned above.

Level specific letter presentation allows us to present in succession two target letters. These may belong to the same or to different hierarchical levels (see Figure 1B). Using a grid as a mask, Lopez et al. (2002) found a large attentional blink at short SOAs in different- trials, which was absent in same-level trials. The attentional blink lasted for about 400 ms for the local/global shift, and more than 1600 ms for the global/local shift. This advantage for local/global shifts could explain the attentional advantage of the global over the local level described for traditional Navon tasks (Kimchi, 2014). Other studies have replicated these results (White et al., 2009; Valdes-Sosa et al., 2014). Note that with level-specific letter presentation we can disassemble the traditional Navon task, controlling the direction of in which attention shifts between levels which is not possible with the traditional Navon task.

### A common theoretical framework

To provide a common conceptual structure for object-based attention and attention to hierarchical levels, we start off with the proposal that compound letters must be perceived via object-files, just like any other visual object. Theories of hierarchical perception have ignored this fact (or have downplayed it, see Robertson, 1996). Interestingly, Hübner and Volberg (2005) provide evidence that letter identity and hierarchical level are represented separately in earlier processing stages, and must be subsequently bound for reporting (at a stage they did not specify). This contradicts the traditional assumption that these aspects are inseparable during initial stages of perceptual processing. This proposal is supported by the fact that subjects make more conjunction errors (i.e., a correct identity from the wrong level) than predicted by this traditional view, particularly when compound letters are presented only briefly. Here we propose that this putative binding of letter and level identities requires the use of an object-file.

Furthermore, we posit that object-files for the different hierarchical levels are independent and have incompatible formats. Treisman (2006) advocated that, depending on the perceptual strategy of the observer, object-files can consist of either single objects, ensembles of objects, or even scenes. Obviously the types of information that these diverse object-files can bind, as well as their internal structure, must be very different. Object-files can mutate their form without losing their continuity (i.e., an open hand clenching into a fist). This implies that different letters can be represented by the same object-file. However, we stipulate that this only happens if they have a compatible level-specific format.

We have seen that spatiotemporal congruity and featural similarity are necessary to maintain object continuity in face of sensory change (Scholl, 2007; Flombaum et al., 2008). Object-file persistence depends also on cohesion. This implies that an object-file cannot survive after splitting into distinct identical objects (Mitroff et al., 2004, 2005a). Attending to local letters implies segregating a whole into a collection of parts (Han and Humphreys, 2002), which would destroy the object-file for the global form. Segregation of different object-files is also required for their persistence. This means if different objects merge, their object-files cannot survive as autonomous entities (Scholl et al., 2001; Mitroff et al., 2005b). This implies that integrating local letters into a global letter (Han and Humphreys, 2002), would eliminate their independent representations.

We propose extending the object-file theory in an additional direction. The creation of a new object-file (attentionally more demanding than its update), should be especially susceptible to the quality of figure/ground segregation (Peterson, 2001; Peterson and Kim, 2001). An object cannot be represented by an object-file until it is parsed from the background (Peterson, 2001). Thus, noise or masking should impair object-file creation, as recently reported by Wutz and Melcher (2013). This deleterious effect of noise and masking should be greater when attention is already invested on an existing object, and thus less available for new object-file creation. Finally we assume that the object-files in our experiments do not survive from one trial to another.

### Overview of this article

Our first goal was to replicate our previous findings with the TSTT with compound letters and based on level specific letter presentation. Thus, we verified if different-level trials elicited a larger attentional blink than same-level trials, while including additional controls for several potential confounds (Experiments 1 and 2). Furthermore, in Experiment 2 we measured the duration of the attentional blinks more precisely by using an adaptive staircase procedure. In this experiment we also confirmed the attentional nature of the inter-target interference by having participants ignore T1 in some trials. In these experiments we used two types of mask (with different spatial frequency content) to curtail the persistence of the targets. Previous results (Valdes-Sosa et al., 2014) suggest that these masks impair T2 recognition differently as a function of the latter's hierarchical level, a finding we confirmed here. In Experiment 3 we explored the nature of this effect by simulating the inattention to T2 produced during the attentional blink by reducing of the contrast of the letters. Finally in Experiment 4, two moving objects were presented, either of which could harbor the compound letter targets. The goal was to directly explore the participation of object-files in attention to hierarchical levels. In this experiment we compared the effects of switching location, hierarchical level, and objects on the attentional blink, as well as their interactions. In the general discussion, we elaborate the extended object-file theory presented here and compare its ability to explain our data with other models concerning hierarchical perception. In all these experiments the level of T1 was forewarned by a cue word. In Experiment 5 we explored the role of endogenous reconfiguration of executive processes (associated with task switching) by manipulating cue validity over trials.

## Experiment 1

In this experiment we replicated and extended previous work with TSTT based on level specific letter presentation (Lopez et al., 2002; White et al., 2009; Valdes-Sosa et al., 2014). The novelty of this approach over traditional Navon figures is that it is possible to separate the presentation of local and global letters by variable amounts of time. Four types of transition between targets were used (global/global, local/local, global/local, and local/global). These were interspersed within the same blocks in a random order, in contrast to the blocked procedure used in our previous work (Lopez et al., 2002). The goal was to verify if the attentional blink was smaller in same-level trials, and larger in different level trials as previously reported.

We also examined if the quality of the masks temporally enclosing T2 was capable of affecting the size of the attentional blinks, as suggested by a previous study (Valdes-Sosa et al., 2014). One mask, containing relatively higher SF (HSF), was the original grid used to generate the compound stimuli (also used as masks by Hübner and Volberg, 2005; Flevaris et al., 2010). The other mask, containing relatively more low SF (LSF) content, was generated by superimposing random line segments on this grid. In Valdes-Sosa et al. (2014), the two masks were used with different participants, a shortcoming corrected here. Furthermore, our previous work used fixed durations for the global and local letters. Since the latter are more difficult to read, uncontrolled differences in readout times between levels could have existed. This could have distorted estimates of attentional blink magnitudes. Here we controlled this factor by separately titrating in each subject the contrasts and durations of the global and the local letters in order to equate their ease of identification. The Quest staircase algorithm was used for this titration (Watson and Pelli, 1983).

### Methods

#### Participants

Ten university graduate students (4 females) from the Cuban Center for Neuroscience, with ages between 25 and 35 years, were recruited for the study. All subjects had normal, or corrected to normal vision, and none had a history of neuropsychiatric disorders, nor were they taking psychotropic drugs at the time of this experiment. A written informed consent was obtained from all participants and the experimental protocol was approved by the ethics committee of the Cuban Center for Neuroscience.

#### Stimuli

**Figure 2:**
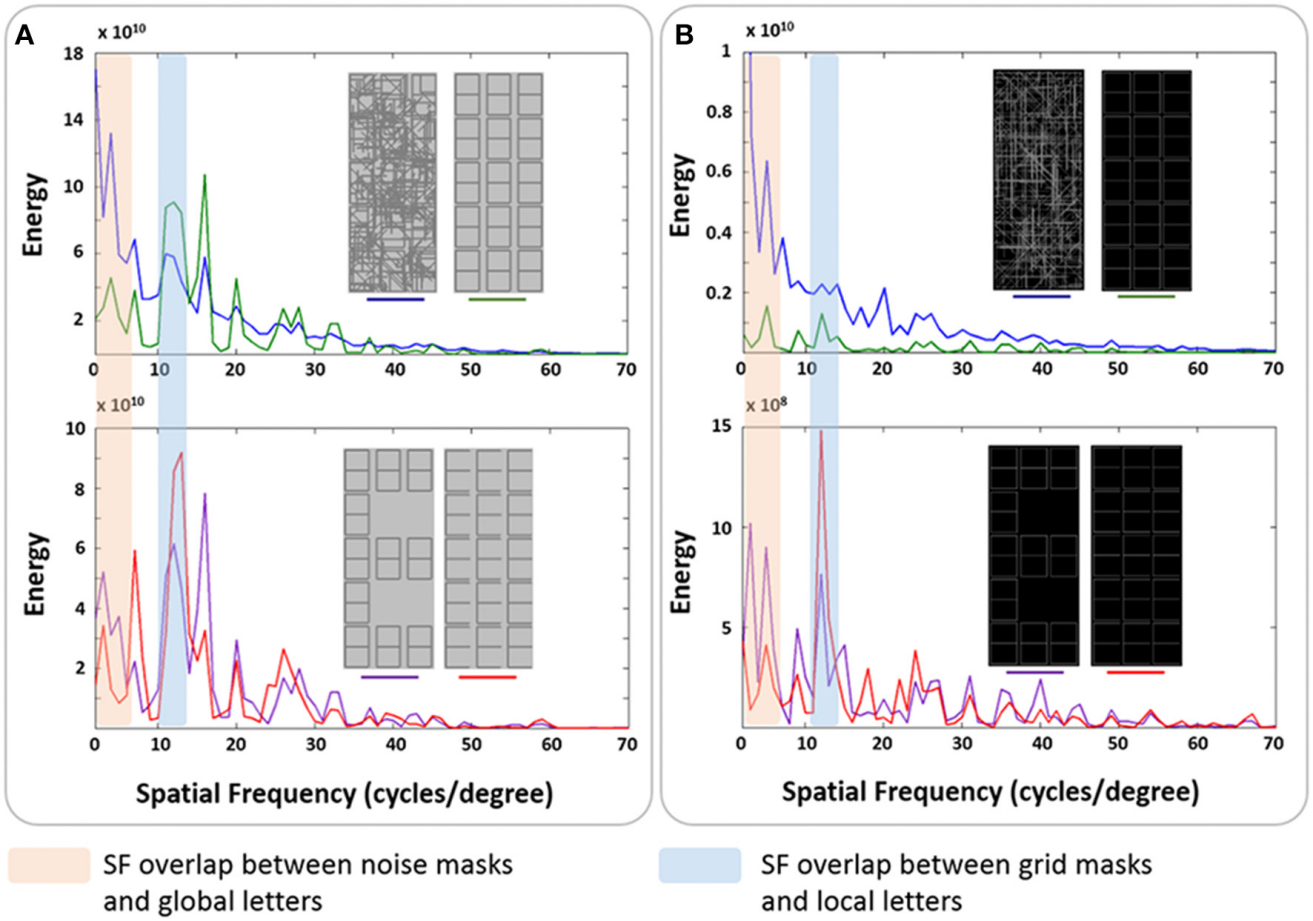
**Spatial frequency spectra of the stimuli from Experiments 1 and 2**. Rotational spectra were obtained for masks and the global and the local letter “E,” as described in the Methods of the article. Areas of coincidence between mask and letter spectra are indicated as mask/letter SF overlap. **(A)** The two mask types used and global and local letter E from Experiment 1. **(B)** The two mask types used and global and local letter E from Experiment 2.

Dark-gray characters (see Figure 1 for a black and white rendering, and Figure 2 for a more realistic gray tone depiction) were displayed on a light-gray background, at the center of a CRT screen placed 40 cm in front of the observers. Letters were obtained by modifying selected segments of a grid that comprised 15 rectangular “8” figures. Five letters were used (E, H, S, P, U), that could appear at either the global or local level. Global letters (see Figure 1) were obtained by increasing the saturation (i.e., making the gray lighter) of selected “8” figures (thus reducing their contrast with the background), and roughly occupied the same area as the original grid, which was 100 mm high and 38 mm wide (approximately 7.2 × 2.43° of visual angle). Local letters (see Figure 1) were obtained by increasing the saturation of individual segments within all the “8” figures, and were 18 mm high and 10 mm wide (approximately 1.42 × 0.8° of visual angle).

The rotational energy spectra of the grid and noise masks, as well as global and local “E,” were calculated. The sfPlot function (based on the 2-D Fourier transform), as implemented in the Shine Toolbox (Willenbockel et al., 2010), was used for this estimation. The resulting spectra were modulated by the corresponding values of the contrast sensitivity function equation described by Mannos and Sakrison (1974). Figure 2A shows the energy spectrum of the two masks and the letters. The noise mask had more energy at LSF than the grid masks. The latter had a large peak at HSF. The spectra of the global and local E respectively overlap best with the noise and grid masks. We will dub this coincidence in spectral peaks as mask/letter SF overlap.

### Titration of letter contrast and duration

The contrast used to produce the global and the local letters was titrated in each subject a separate session (before the main experiment) with a Quest staircase (implemented on Matlab 6.5, Mathworks Inc., see Watson and Pelli, 1983), using a 75% correct recognition threshold. Trials consisted of a randomly selected letter presented for 150 ms, preceded and followed by a mask, both of which lasted 300 ms. The initial Quest parameters were: beta = 3.5, delta = 0.01, gamma = 0.5, and grain = 0.01. Titration was performed separately when using the grid or the noise masks. Letter duration was subsequently titrated with the same staircase. Note that in the other experiments of this article only letter duration was titrated. Mask contrast was not titrated.

### Procedure

Two blocks of trials were used, one with the grid mask and the other with the noise mask, with the order counterbalanced across subjects. The nature of T1 was forewarned by the words “GLOBAL” or “LOCAL” at the beginning of every trial. Participants then triggered the events presented in Figure 1B by pressing the spacebar on the computer keyboard. The initial mask was presented and after a 300 ms delay, it was first briefly substituted by T1, which could be either a global letter or a set of local letters. The letter was then replaced by the inter-target mask. After a delay, the second target (T2) was briefly revealed and then replaced by the final mask. Four stimulus onset asynchrony (SOA) values were used: 200, 400, 800, and 1600 ms. At the end of each trial, the observers reported (in a forced-choice) both the T1 and T2 letters. According to the level of the two targets, two types of same-level trials (global/global and local/local), and two types of different-level trials (global/local and local/global) were used. These 4 transitions types were presented randomly mixed in a single block. T2 accuracies (only from trials with correct T1 recognition were used in this article) was submitted to repeated measures ANOVA, using three within-subject factors: Mask-type (grid vs. noise), Transition-type (global/global, local/local, global/local, and local/global) and SOA (200, 400, 800, and 1600 ms). The Greenhouse-Geisser correction was used when appropriate in these and in all subsequent analysis (Greenhouse and Geisser, 1959).

### Results and discussion

The mean titrated durations for all stimulus types are shown in Table 1. They were significantly [*F*_(1, 9)_ = 112.2, *p* < 0.0001] shorter for global letters (60.1 ms) than for local letters (122 ms), and significantly shorter [*F*_(1, 9)_ = 9.6, *p* < 0.012] for grid masks (75 ms) then for noise masks (107 ms). The interaction between these effects was not significant.

**Table 1:** **Mean titrated durations of letters in Experiment 1**.

	**Global**	**Local**	**Average**
Grid	52.8	97.2	75.0
Noise	67.4	146.8	107.1
Average	60.1	122.0	

**Figure 3:**
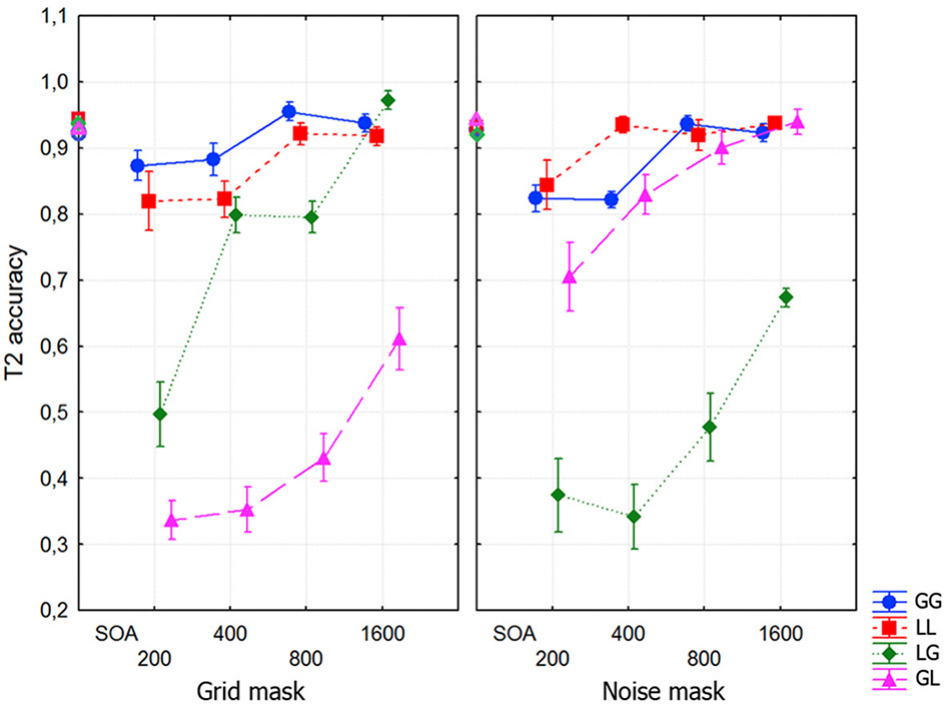
**T2 accuracy as a function of Mask-type, Transition-type and SOA**. In these and all subsequent graphs, means and standard errors of targets are plotted, and for T2 accuracy calculation only trials with correct T1 identification were included. Note that the corresponding T1 accuracies are plotted at SOA zero. In this figure and the rest the following acronyms are used: GG (global/global), LL (local/local), LG (local/global), and GL (global/local).

Recognition of T1 was accurate for all types of trial in every subject (>85%, see Figure 3). The mean T2 accuracies as a function of Mask-type, Transition-type and SOA are shown in Figure 3. The Mask-type effect was not significant. Transition-type was highly significant [*F*_(3, 27)_ = 130.5, *p* < 0.0001, η^2^ = 0.35], and SOA was also highly significant [*F*_(3, 27)_ = 93.046, *p* < 0.0001, ε = 0.586, η^2^ = 0.12]. The interactions between Mask-type and Transition-type [*F*_(3, 27)_ = 224.720, *p* < 0.0001, ε = 0.699, η^2^ = 0.32], between Transition-type and SOA [*F*_(9, 81)_ = 9.8462, *p* < 0.0001, ε = 0.416, η^2^ = 0.042] and between Mask-type, Transition-type and SOA were all highly significant [*F*_(9, 81)_ = 6.7418, *p* < 0.001, ε = 0.372, η^2^ = 0.024]. Planned comparisons showed that T2 accuracy was larger in same-level than in different-level trials (*p* < 0.0001).

An additional ANOVA, including only the same-level trials, exhibited a highly significant SOA effect [*F*_(3, 27)_ = 19,813, *p* < 0.0001, ε = 0.665, η^2^ = 0.26]. For the two masks, accuracy was significantly lower than the maximum (at 1600 ms) at 200 and 400 ms (each *p* < 0.0001). The interaction between Mask-type and Transition-type was also significant [*F*_(1, 9)_ = 6.1132, *p* < 0.04, ε = 1.0, η^2^ = 0.057]. This was due to a higher T2 recognition accuracy in global/global, compared to local/local trials only for the grid mask (*p* < 0.016).

Another ANOVA was performed including only the different-level trials. The effect of Mask-type was significant [*F*_(1, 9)_ = 6.378, *p* < 0.04, η^2^ = 0.014], with slightly lower T2 accuracies for noise compared to grids. Transition-type was not significant, whereas SOA was highly significant [*F*_(3, 27)_ = 95.769, *p* < 0.0001, ε = 0.932, η^2^ = 0.234]. The interaction between SOA and Mask-type was also significant [*F*_(3, 27)_ = 4.829, *p* < 0.0081, ε = 0.929, η^2^ = 0.009]. Importantly, the interaction between Mask-type and Transition-type was highly significant with a strong effect [*F*_(1, 9)_ = 747.63, *p* < 0.0001, ε = 1.0, η^2^ = 0.54]. This interaction was further analyzed with planned comparisons. With grid masks the attentional blink was significantly smaller in local/global than in the global/local trials (*p* < 0.0001). The opposite pattern was found for noise masks (*p* < 0.0001). Finally, the interaction between Mask-type, Transition-type and SOA (see Figure 3) was highly significant [*F*_(3, 27)_ = 9.2549, *p* < 0.001, ε = 0.757, η^2^ = 0.03]. This effect was due to a faster recovery of the attentional blink for local/global trials and a much slower recovery in global/local trials with grid masks, with the opposite pattern for noise masks. We did not observe in this, and subsequent, experiments any level/identity binding errors (which would have resulted in reporting the targets in the wrong order in different-level trials).

In this experiment, we used level specific letter presentation to eliminate ambiguity about letter hierarchical level (which precluded level/identity binding errors), while carefully equating the difficulty of global and local letter recognition ease. Also, trial types were randomly interspersed within the same blocks, thus avoiding long term biases that could have resulted from grouping transition types into blocks (as in Lopez et al., 2002; White et al., 2009). Finally, both grid and noise masks were used within the same participants (in contrast with Valdes-Sosa et al., 2014). With these additional controls, we were able to replicate and extend our previous findings.

Small -albeit significant- attentional blinks were found for same-level trials. This effect is perhaps similar to that found in mainstream attentional blink research (Dux and Marois, 2009). Much larger attentional blinks were replicated for different-level trials, that were relatively shorter (approximately 0.5 s) for local/global trials but much longer trials for global/local trials when grid masks were used (>1 s). The opposite pattern was seen across different-level trials when noise masks were used. This confirmed the findings from Valdes-Sosa et al. (2014), but now with a statistically significant within-subject interaction between Mask-type and T2 level for different-level trials. No evidence for a lag-1 effect was found, although shorter SOAs than those used here are needed to exclude this possibility.

Only a few studies have studied the attentional blink produced by traditional compound-letters. One series of studies (Lawson et al., 1998, 2005; Crewther et al., 2007) presented multiple distracters in addition to a T1 distinguished by color, and a pre-designated symbol as T2. They found unusually long attentional blinks, lasting from 1.5 to 2 s, for all types of transitions. Puzzlingly, there was no reduction of the attentional blink in same level trials. Their task may have been more difficult than ours due to the use of many distracters. Also, since there was no advance knowledge of T2 level, extra time may have been needed to resolve this uncertainty and also to resolve the response conflict inherent to traditional Navon figures. By using level specific letter presentation, we were able to avoid both problems.

Findings closer to ours were reported by Srivastava et al. (2010). They used TSTT with traditional Navon figures, with the targets placed at two out of four spatial locations followed by visual noise masks. The levels of T1 and T2 were pre-specified at trial start, but the location was forewarned only in their second experiment. In both experiments, same-level trials elicited very small attentional blinks, similar to our results. However, they did not find any difference in attentional blink magnitude between global/local and local/global trials in Experiment 1. This discrepancy could have arisen from the unpredictable T2 location in this experiment. Their Experiment 2 eliminated spatial uncertainty, and elicited larger attentional blinks for local/global than for global/local trials, more in line with our findings with the noise masks. Again the use of level specific target presentation probably allowed us to obtain cleaner estimates of attentional dwell times.

How can we explain the large attentional blinks observed here for different-level trials which are absent for same-level trials? One possibility is the “attentional print” posited by Robertson (1996). She hypothesized that identification of a letter within a traditional Navon figure creates this print, which attracts attention to features typical of its hierarchical level. This could be achieved by facilitation of specific SF bands (see also Flevaris et al., 2010). In other words, stimuli would enduringly bias competition between SF bands in favor of their dominant spectral content. This model explains level-specific priming with traditional Navon figures, which consists of faster identification of a letter in one trial when it is presented at the same (relative to a different) level as in the previous trial (Robertson, 1996; Kim et al., 1999). This type of priming presents interesting analogies with our data. However, level-specific priming has a long duration (in the order of several seconds) and is carried over between different trials. Our same-level facilitation (reflected by an absence of an attentional blink) occurs within the same trial and is present at much shorter time intervals (in the order of hundreds of milliseconds). We will return to the relationship between the two phenomena in the next experiment. Nevertheless, an attentional print as described by Robertson, could certainly produce the pattern of attentional blinks described in this experiment. This would be a form of feature-based selection.

On the other hand, our extended object-file theory also accommodates all the data from this experiment. If T2 is at the same level as T1 it can be assimilated as an update of the corresponding (recyclable) object-file. This is attentionally undemanding, therefore the attentional blinks should be small for this type of trials. In contrast, a new object-file must be created for T2 on different-level trials, which is attentionally very demanding. Since T1 sequesters attention, this entails large attentional blinks for this type of trial. The effects of Mask-type on T2 recognition would be mediated by their deleterious effect on object-file formation, which would interact with the impoverished attention existing at T2 presentation. Note that T2 recognition was most impaired for larger mask/letter SF overlaps. These assumptions were tested more directly in Experiment 3. Note that the attentional print and object-file accounts do not mutually exclude each other, and both make identical predictions about Experiment 1.

## Experiment 2

The previous experiment showed that, at short SOAs, it was difficult to divide attention over between successive compound letters if they occurred at different hierarchical levels. Furthermore, this difficulty was more pronounced when the mask/letter SF-overlap for T2 was larger. In the present experiment we aimed at reproducing these findings with the following extensions. First, we tested if the large attentional blink found in different-level trials was reduced when T1 was disregarded by the observers. This allowed us to assess how much of the T2 impairment was due to sensory interactions and how much due to attentional factors. Second, the same test was used to determine if the effect of Mask-type on attentional blink size found in Experiment 1 also depended on attention. Valdes-Sosa et al. (2014) found a reduced attentional blink with grid masks when T1 was ignored, but did not examine this with noise masks. Therefore, they did not statistically test the interaction of Mask-type with attention in a within-subject design.

Furthermore, we were interested in obtaining more precise estimates of the duration of the attentional blinks. To achieve this we used a staircase psychophysical procedure linking inter-target interval to accuracy in T2 identification (Watson and Pelli, 1983). Note that although we report attentional blink durations as SOA units, as is standard in the literature, we also analyzed the inter-stimulus durations (ISIs) to take advantage of the procedure for titration of letter readout times introduced in Experiment 1. Remember that this allowed us to adjust the duration of letter presentations in order to compensate the slower readout of the local- relative to the global-level. If we assume that letter readout times were held constant, then the ISI are a purer comparison of attentional dwell times. The staircase procedure had the additional advantage of reducing testing to a tolerable duration, since we needed to compare attentional blink durations within the same participants while varying trial types, masks, and attend/ignore-T1 conditions.

Another change respect to Experiment 1 is that the contrast of all stimuli was reversed, and the energy of the noise mask increased, to see if these physical characteristics of the stimuli had any effect on attentional blink durations. Finally a control was introduced to examine if global/local selection could be based on zooming in attention (Stoffer, 1993) to a few selected locations in the visual field (e.g., near fixation). Here for each local target, letters were only unveiled ata subset of locations within the stimulus matrix. Since these locations were randomly selected for each target, a strategy of monitoring of only a few pre-selected placeholders would have made accurate identification of local letters very difficult.

### Methods

#### Participants

Ten (6 females) university graduate students from the Cuban Center for Neuroscience, with ages between 25 and 35, were recruited for the study. Six of them also participated in other experiments reported in this article. The inclusion criteria and ethics approval were similar to Experiment 1.

#### Procedure

The procedure and trial structure used here were identical to those of Experiment 1 (see Figure 1), except for four critical modifications. First, an inverse contrast to that used in Experiment 1 was employed for the grid, with light-gray characters on a black background (see Figure 2 for gray tone depictions). Second, the letters were defined by decreasing the saturation of the selected line segments (i.e., blacking out). The stimulus duration was previously titrated as described in Experiment 1. Third, local letters were displayed in only 10 out of 15 available positions, which were randomly chosen to discourage attention on a predetermined local element. Finally, we used the same staircase that titrated letter duration but in this case to determine the inter-stimulus interval (ISI) between targets necessary to achieve 75% correct identification of T2. First, an attend T1 block was performed. In this case, the titration was performed only if T1 was correctly reported. Then a separate block, ignoring T1 and only identifying T2 was performed. The ISI thresholds were also transformed into stimulus onset asynchrony (SOA) measures (using the titrated durations for each participant). The ISIs and SOAs were submitted to separate repeated-measures ANOVAs, using three within-subject factors: Mask-type (grid vs. noise), Transition-type (different-level vs. same-level transitions) and T2-level (Global vs. Local). We also compared the attend–T1 and ignore-T1 thresholds for all trial types. The rotational energy spectra of the masks, as well as global and local “E,” were calculated as in Experiment 1, which are exhibited in Figure 2B. The noise mask had more energy at the lowest spatial frequencies than the grid mask, and their spectra respectively overlap best with those of the global and local letters.

### Results and discussion

The mean titrated durations were 54.6 ms for global letters and 114.6 ms for local letters [*F*_(1, 9)_ = 180.9, *p* < 0.0001], and about 79.4 ms for grid masks and 89.9 ms for noise masks [*F*_(1, 9)_ = 8.6, *p* < 0.017] (see detailed durations for global and local stimuli with grid and noise masks in Table 2). The interaction between these effects was not significant.

**Table 2:** **Mean titrated durations of letters in Experiment 2**.

	**Global**	**Local**	**Average**
Grid	50.1	108.6	79.4
Noise	59.0	120.6	89.9
Average	54.6	114.6	

### ISI thresholds

**Figure 4:**
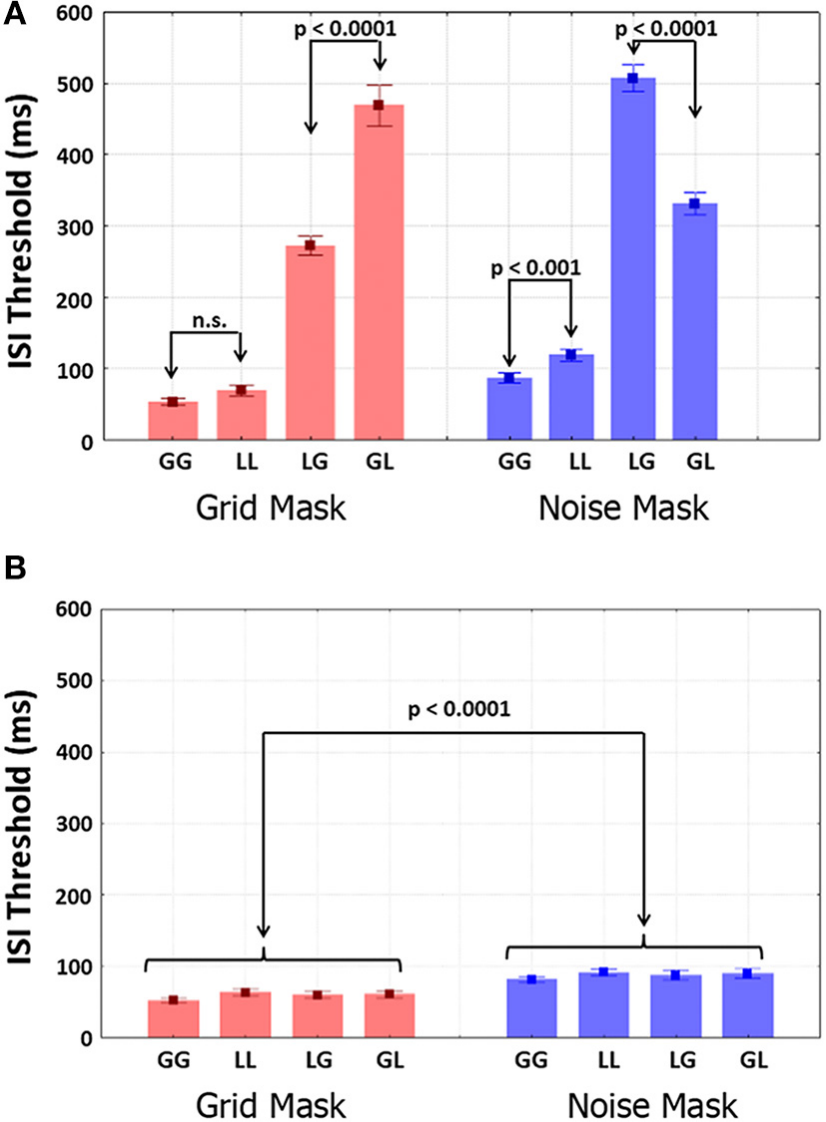
**ISI thresholds obtained with the Quest staircase (for 75% correct T2 identification) for all four possible transitions with the two Mask-types**. **(A)** Results from the attend-T1 test; **(B)** results from the ignore-T1 test. Selected results from the planned comparisons are shown.

Group mean ISI thresholds are shown as a function of Mask-type and trial type in Figure 4A. Mask-type was significant with smaller thresholds for grids in than noise [*F*_(1, 9)_ = 20.692, *p* = 0.001, η^2^ = 0.026]. Transition-type was highly significant, with smaller same-level than different-level thresholds [*F*_(1, 9)_ = 812.87, *p* < 0.001, η^2^ = 0.76]. T2-level was not significant. Importantly, the interactions between Mask-type and T2-level [*F*_(1, 9)_ = 110.97, *p* < 0.001, η^2^ = 0.062] and between Mask-type, Transition-type and T2-level (Figure 4) were highly significant [*F*_(1, 9)_ = 65.608, *p* < 0.001, η^2^ = 0.076]. Planned comparisons showed that global/local thresholds were larger than for local/global transitions with grids (*p* < 0.001). Conversely, local/global thresholds were larger than global/local thresholds (*p* < 0.001) with noise. Thresholds did not differ between global/global and local/local shifts with grids. The local/local were larger (*p* < 0.001) than global/global thresholds with noise. When the participants ignored T1, all the thresholds were very small (Figure 4B), below 100 ms. Only Mask-type was significant, with grid shorter than noise thresholds [*F*_(1, 9)_ = 71.1, *p* < 0.0001, η^2^ = 0.23].

### SOA thresholds

**Figure 5:**
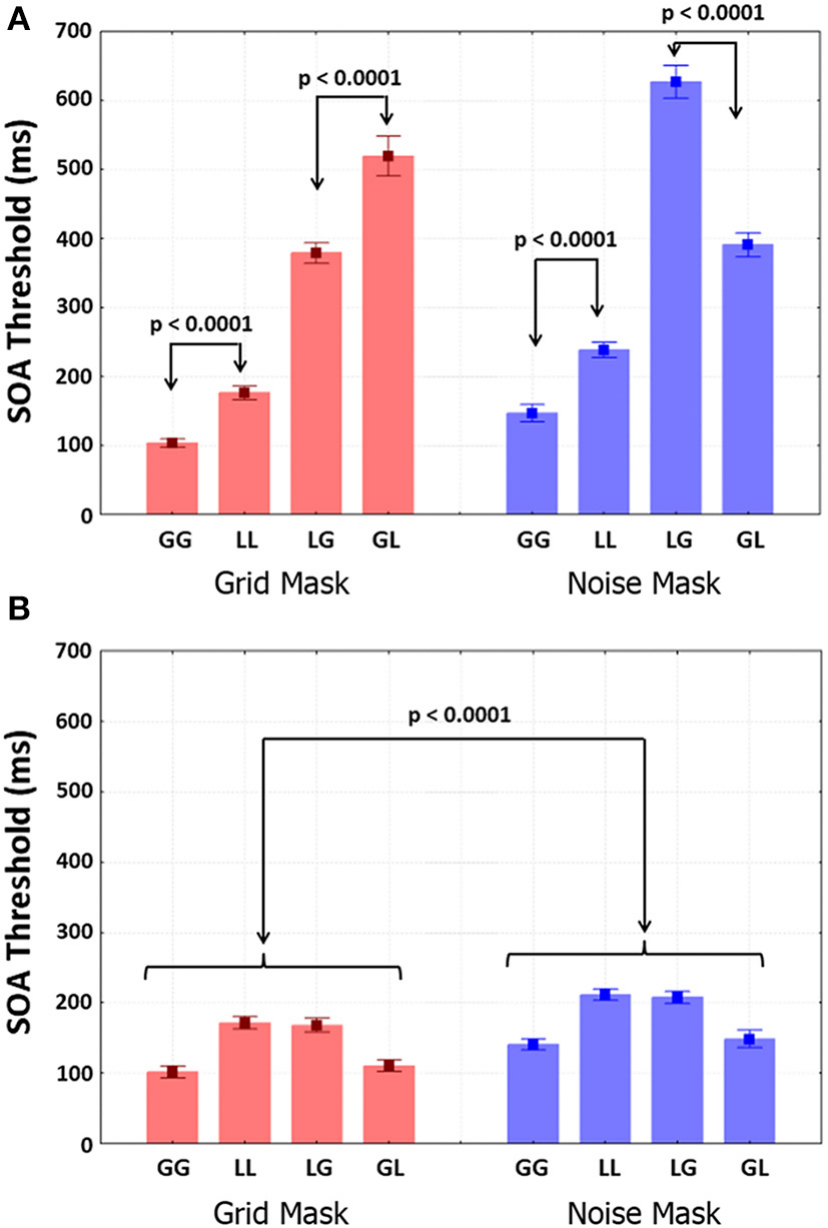
**SOA thresholds obtained with the Quest staircase (for 75% correct T2 identification) for all four possible transitions with the two Mask-types**. **(A)** Results from the attend-T1 test; **(B)** results from the ignore-T1 test. Selected results from the planned comparisons are shown.

Group mean SOA thresholds are shown as a function of Mask-type and trial type in Figure 5A. Shorter thresholds were found for grids than noise [*F*_(1, 9)_ = 32.485, *p* < 0.001, η^2^ = 0.025]. Transition-type was highly significant, with shorter same-level than different-level SOAs [*F*_(1, 9)_ = 812.87, *p* < 0.0001, η^2^ = 0.76]. T2-level was not significant. Importantly, the interactions between Mask-type and T2-level [*F*_(1, 9)_ = 110.97, *p* < 0.001, η^2^ = 0.062], between Transition-type and T2-level [*F*_(1, 9)_ = 64.756, *p* < 0.001, η^2^ = 0.033] and between Mask-type, Transition-type and T2-level (Figure 5A) were highly significant [*F*_(1, 9)_ = 73.305, *p* < 0.001, η^2^ = 0.076]. Furthermore, in an ANOVA restricted to different-level trials the interaction between Mask-type and Transition-types was highly significant [*F*_(1, 9)_ = 95.553, *p* < 0.0001, η^2^ = 0.71]. Longer global/local than /global SOA thresholds were found for grids (*p* < 0.001), whereas longer local/global than global/local thresholds were found for noise (*p* < 0.001). For both mask types, thresholds were larger in local/local than global/global trials (*p* < 0.001).

Thresholds were smaller in the ignore-T1 (Figure 5B) than the attend-T1 conditions. Ignore-T1 thresholds with the grid mask were shorter than with the noise mask [*F*_(1, 9)_ = 68.923, *p* < 0.0001, η^2^ = 0.23], and were also larger for local/local and local/global than for global/global and global/local values for both mask types (*p* < 0.0001). Importantly, thresholds for same-level trials did not differ between the attend-T1 and ignore-T1 sessions. However, in different level trials thresholds for attend-T1 were significantly longer than for ignore-T1 (*p* < 0.0001 in both mask types).

In this experiment we replicated the principal results of Experiment 1 (some quantitative discrepancies are discussed below) using estimates of attentional blink duration obtained with an adaptive psychophysical procedure, instead of error rates from the method of constant stimuli used before. Different-level, but not same level, trials presented long attentional blinks, which were even longer when the mask/letter SF-overlap was increased (with the grid for global/local trials and with the noise mask for local/global trials). Thus, these effects are robust across dissimilar experimental conditions, and for both SOA and ISI measures of attentional blink duration, and did not depend on the direction of stimulus contrast, which was reversed in the two experiments.

The flawless report of local targets in same-level trials, even at very short ISIs, and despite abrupt and random changes in the location of these stimuli, is inconsistent with a zoom lens model. This model assumes that hierarchical level is selected by the size of the attentional window (small for local letters and large for global stimuli; Stoffer, 1993). With our brief masked presentation of local letters, identification would be successful only if the narrow beam of attention could focus in advance at a few placeholders. Alternatively, recognition could succeed if the local letters captured attention fast enough to fit into our estimate of total local letter readout time, while leaving time for letter identification. The first condition was not possible because the location of local letters was unpredictable. And the total local readout time was estimated to be about 114 ms, which would not leave enough time for exogenously triggered covert shifts of attention plus letter identification itself. These shifts consume about 100 ms (see Chakravarthi and VanRullen, 2011). This suggests that local letters are processed in parallel without the need for a narrow spotlight of attention.

Importantly, the instruction to ignore T1 completely eliminated the attentional blink in the different-level trials, as well as the modulations that the Mask-type produced on this effect. The latter interaction was highly significant in a statistical test. This confirms that all of the observed impairments in T2 were truly attentional in nature. Note that if we assume that the titration of letter duration was effective in equating different letter readout times, the ISI estimates assess the time to shift attention between targets. These times were very short (all about 50 ms) when T1 was ignored. Attending T1 in the same-level trials increased these times only slightly. For different-level trials these durations were longer, about 300 ms for the mask with least spectral overlap with the corresponding letter level, and about 500 ms when the spectral overlap was larger. This suggests that Mask-type can delay availability of T2 for recognition for about 200 ms.

The estimates of attentional blink durations in this experiment were shorter than those of Experiment 1. Here the threshold for T2 accuracy was achieved at a SOA of about 600 ms in the worst cases. In Experiment 1, the same threshold was achieved much later for the worst cases; with impairments lingering up to 1600 ms. This suggests that in Experiment 1 we really observed two components of the attentional blink: a first shorter-lasting and mandatory component (that was also present in Experiment 2); and a second longer-lasting and optional component. The latter component is in the same time frame as level-specific priming described for traditional Navon figures (several seconds, see Robertson, 1996). Such long effects are not common in the attentional blink literature. However, Lawson et al. (1998) using compound figure in an attentional blink paradigm very different from ours also found a different level effects that lasted several seconds. Thus, level-specific priming may not be completely equivalent to the level switch effects observed in our paradigm.

## Experiment 3

Experiments 1 and 2 both confirmed that the attentional blink typical of different-level trials was markedly affected by masks temporally enclosing the targets. The impairment was worse when T2 mask/letter SF overlap was largest, which occurred in LSF regions for global letters and the noise mask and in HSF regions for local letters and the grid mask. Experiment 2 indicated that mask/letter SF overlap could delay recognition of these attentionally blinked T2s by about 200 ms, but did not delay recognition of the T2s from same-level trials, nor of T1 in any condition. How does Mask-type exert its effect on T2 recognition? Here we examine two logical alternatives. Mask-type could act either on processes that are triggered by T1 or those triggered by T2.

An example of the first sort of process would be the generation of attentional settings by T1. The attentional print proposed by Robertson (1996) could play this role. In principle, the SF selectivity of such a print could interact with spectral content of the mask producing an effect on T2 recognition. Although this view seems straightforward, in the discussion of this experiment we see how it runs into trouble with our data. The second sort of process (triggered by T2 presentation) could involve object-file creation. This would be consistent with research by Wutz and Melcher (2013) who examined the effect of masks on different stages of object-file formation. They found that forward (“integration”) masking selectively impaired object individuation, whereas backward (“interruption”) masking hampered object identification as well as consolidation of information into visual working memory. Both forward and backward masking were present in our experiments, and we have postulated that object file creation is specifically needed in different/level trials. On this view, mask/letter SF overlap would slow down the creation of an object-file for an attentionally impoverished T2 stimulus.

These two hypothetical loci for Mask-type effects (T1 attentional prints and speed of T2 object-file formation), are difficult to disentangle experimentally within the TSTT paradigm. The attentional blink was large only in different-level trials, in which both an adverse attentional print and the need for a new object-file would afflict T2. In the present experiment we examined the effect of Mask-type on object-file formation while trying to mimic T2 attentional deprivation but eliminating T1 (in order to preclude generation of an attentional print). Attentional deprivation was necessary since we know that attentionally privileged T1s are immune to mask effects, and that restoring attention to T2 (by ignoring T1) has the same outcome. To simulate the effects of attentional deprivation we reduced the contrast of single targets without affecting that of masks. Previous research (Carrasco et al., 2000, 2004; Cameron et al., 2002) has documented that withdrawing attention decreases the effective contrast of visual stimuli. If Mask-type influences object-file formation depending on attention and mask/letter SF overlap, we can predict the direction of shifts in the psychometric function relating identification accuracy to contrast. The noise mask should increase the contrast thresholds for global letters, whereas the grid mask should increase thresholds for local letters.

### Methods

A group of 9 participants (6 females) were recruited with the same criteria used in Experiment 1. Their ages ranged from 26 to 33 years. Five of them also participated in other experiments reported in this article. They were asked to identify the same five different letters from Experiment 1 (also with the same screen dimensions and visual angles), which could appear at either the global or the local level. The letters were obtained from the 15 “8” grid (Figure 1) by modifying selected line segments, as in Experiment 1. However, here, the contrast of these segments, which defined the letters, was roughly varied in 8 equidistant steps, ranging from almost no change of the original grid to a clearly visible letter (see examples in Figure **7**). The trial structure was as follows. Participants initiated trials by pressing the spacebar on the computer keyboard. The mask from one paradigm was presented for 300 ms. Subsequently, a letter with variable contrast was presented. The same mask was then presented for an additional 300 ms. At the end of the trial, participants were required to indicate the identity of the letter by pressing the corresponding a key on the computer keyboard (a 5 alternative forced choice). As in previous experiments, letter durations for each level were individually titrated in a separate session. The grid and noise masks were employed in separate blocks (the order of which was counterbalanced across subjects). Ten trials per letter and contrast value were presented at each hierarchical level.

For all conditions, logistic curves were fitted to the discrimination accuracies as a function of letter contrast in each participant (using the Palamedes toolbox, www.palamedestoolbox.org, with a guess rate = 0.2, and a lapse rate = 0). The estimated thresholds (75% accuracy), and slopes, of these curves were submitted to a repeated measures ANOVA with two within-subjects factors: Mask-type (grid vs. noise) and Letter-Level (global vs. local).

### Results and discussion

**Figure 6:**
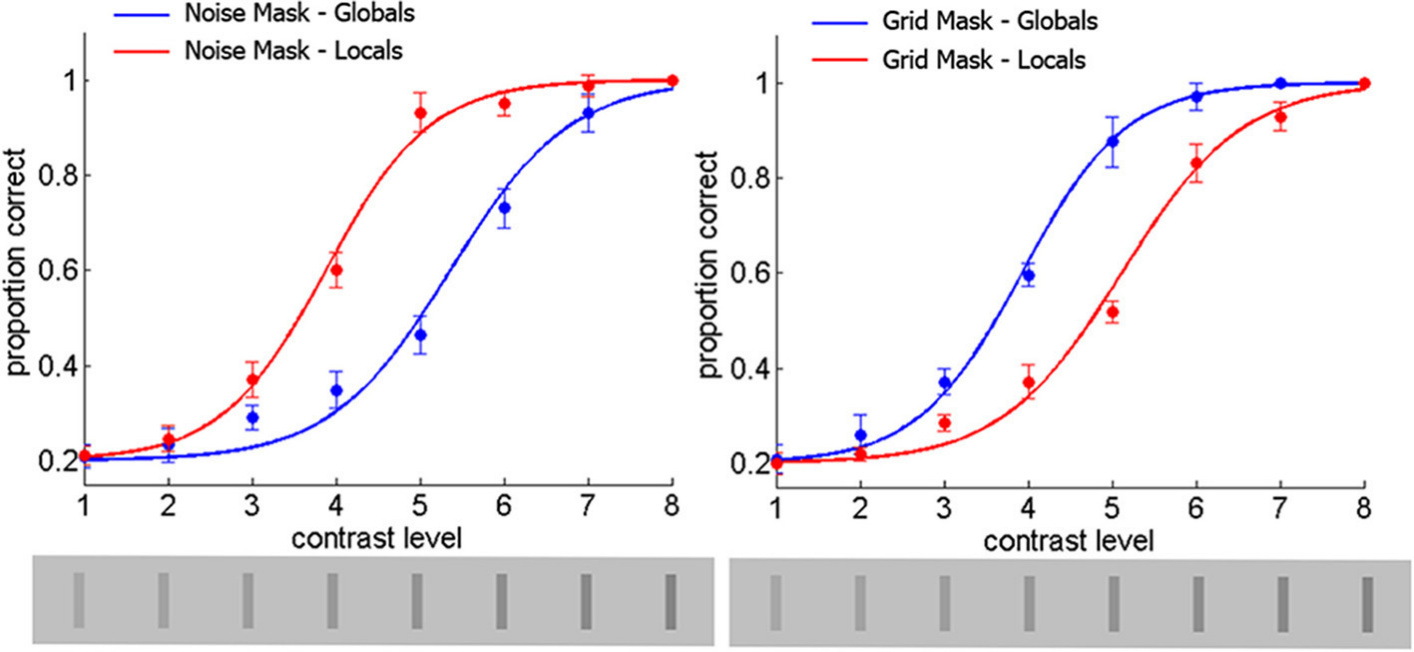
**Statistics for slopes of the contrast psychometric curves estimated in Experiment 3 as a function of Mask-type and letter level**.

Logistic curves for each condition were constructed with the average of the group thresholds and slopes and are exhibited in Figure 6. Statistics of the raw data are overlaid on these curves. The curve for the global letters was shifted to the left (lower contrast thresholds) relative to that of the local letters for the grid mask. Conversely, the curve for the local letters was shifted to the left relative to that of the global letters for the noise mask. These effects were verified in the ANOVA described below.

**Figure 7:**
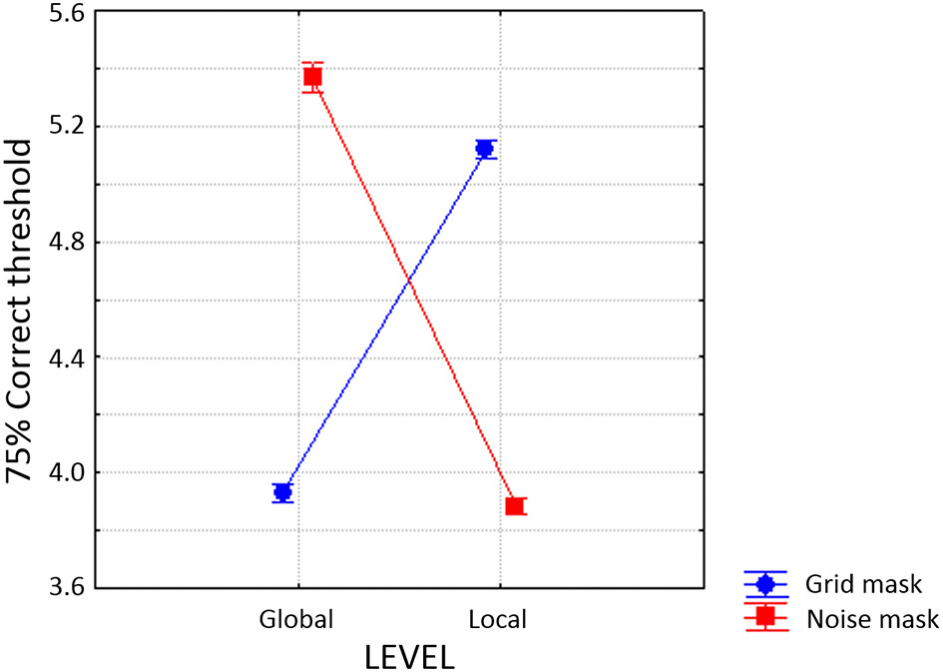
**Discrimination of masked letters as a function of Mask-type and Letter-Level from Experiment 3**.

The effect of Mask-type on the thresholds was significant [*F*_(1, 8)_ = 10.4, *p* < 0.012] due to slightly larger values for the noise mask, although with a weak effect size (η^2^ = 0.0054). The effect of Letter-Level was also significant, [*F*_(1, 8)_ = 23.6, *p* < 0.0013] due to slightly larger values for the global level, with a weak effect size (η^2^ = 0.0.0118). The Mask-type x Letter-Level was highly significant [*F*_(1, 8)_ = 2310.0, *p* < 0.00001] with a very large effect size (η^2^ = 0.9713). This interaction was due to a crossover of the threshold means as can be observed in Figure 7. Planned comparisons showed that all the cells were different in this ANOVA (*p* < 0.001).

In the ANOVA for slopes, neither Mask-type, nor Letter-Level produced significant effects, [both *F*_(1, 8)_ < 1]. The interaction of Mask-type X Letter-Level was significant [*F*_(1, 8)_ = 6.61, *p* < 0.0331], with a moderate effect size (η^2^ = 0.2237). This interaction was due to a crossover of slope means (see Figure 7). Planned comparisons showed that with the grid mask the slopes were significantly larger for the global than the local letters, and that for the global letters the slope was larger for the grid than the noise mask (both *p* < 0.05).

The results show that Mask-type influences letter recognition in the predicted directions: noise increase contrast thresholds for global letters, whereas grids increase contrast thresholds for local letters. This is consistent with the proposal that different spatial frequency bands are used in representing global (relatively LSF) and local (relatively HSF) levels of compound letters (Shulman et al., 1986; Shulman and Wilson, 1987; Robertson, 1996). If we assume that contrast reduction is a proxy for inattention (Carrasco et al., 2000, 2004; Cameron et al., 2002), then the influence of Mask-type on T2 recognition could be explained without the need to invoke attentional settings generated by T1. With focused attention (equivalent to a large effective contrast) performance is asymptotically accurate, thus the effect of Mask-type is not observable. This would be the situation for T1, T2 updates (i.e., same-level-trials), or T2 formation at long SOAs from T1. With reduced attention (medium effective contrast) performance is within the range for which Mask-type effects can be observed. This would be the situation for T2 formation (different-level trials) at short SOAs from T1.

At first glance, the distinct spatial frequency profiles of the two masks suggests that they exert their effects though a attentional print analogous to that posited by Robertson (created by T1). However, further consideration suggests that this explanation does not really fit our data. Suppose that T1 generates a bias toward one SF band. To produce an effect on T2, the trailing inter-target mask would have to either increase or decrease this bias (i.e., noise masks would favor a LF band, and grid masks would favor a HF band). This adjustment of the bias would not depend on the level of the subsequent T2. Among other predictions that do not match our findings, this would imply larger attentional blinks for same-level trials when the spectral content of T2 and mask do not match relative to when it does. This was not observed. Of course, co-opting extra assumptions to an attentional print model could perhaps solve these discrepancies in the future.

Thus, we have shown that an attentional print is not necessary to produce an effect of Mask-type on T2 recognition (although this does not preclude its contribution in Experiments 1 and 2). The most parsimonious explanation of our results is that increasing the amount of mask/letter SF overlap slows down the object-file formation needed to handle the T2 in different-level trials. This affects a process that is already vulnerable to competition from T1, since formation of an object-file is more attention- greedy than its update. Both object individuation, identification, and consolidation into visual working memory could have been modulated since we used both forward and backward masking (Wutz and Melcher, 2013). This interpretation is consistent with the well-known role of critical bandwidths of spatial frequency in the identification of printed letters of different sizes (Chung et al., 2002; Majaj et al., 2002).

## Experiment 4

We have interpreted the results of previous experiments using object-file theory. However, they may also understood (partially) by a feature selection model based on SF channels. Most work on hierarchical perception has ignored or discarded object-based attention. In her discussion of level-specific priming Robertson (1996), recognizes that it shares some similarities with the phenomenon of preview benefits. With preview benefits, a letter reappearing on the same object-file is recognized faster than if it appears on a different object-file (Kahneman and Treisman, 1984; Kahneman et al., 1992). However, she argues that level-specific priming is not related to object-files. Nevertheless, hierarchical perception has not been tested with stimuli specifically designed to evince object-files properties, such as moving objects that compete for attention.

The stimuli used in our previous experiments occupied the same locations, had ambiguous object membership, and varied in only one feature: hierarchical level. In the present experiment, we directly pitted the possible effects of spatial-based, feature-based, and object-based attention against each other. This was achieved by presenting two visual objects that first approachedeach other, then coincided at one point in space (under an occluding figure), only to move apart afterwards. This separation phase can be perceived either as objects that slide past each other, or alternatively as objects that bounce apart. Although sliding is typically perceived more frequently (Bertenthal et al., 1993; Sekuler and Sekuler, 1999), this preference changes to bouncing if the objects swap a feature (such as color) under the occluding figure (Feldman and Tremoulet, 2006; Kawachi et al., 2011).

**Figure 8:**
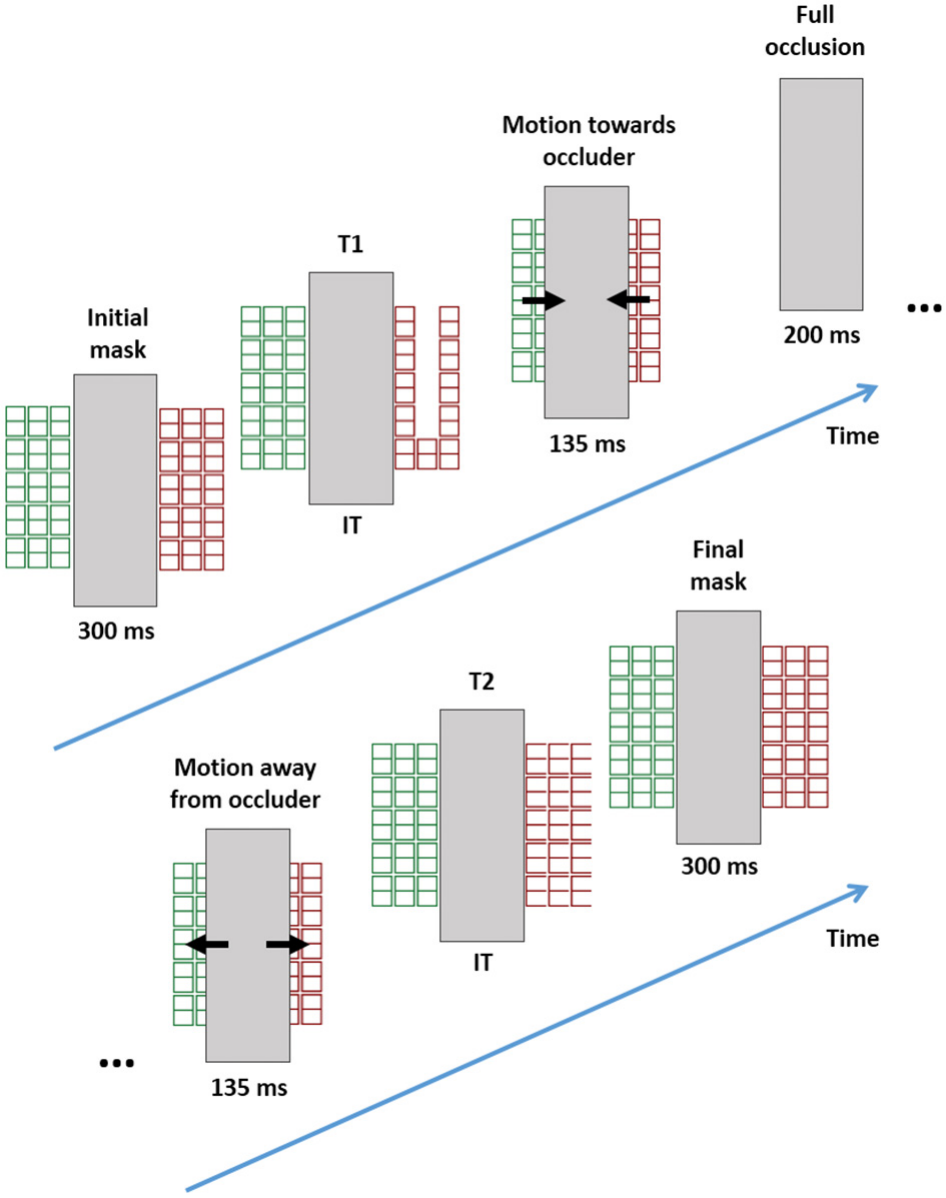
**Stimuli and trial structure in the Experiment 4**. The example is a global/local transition.

This setup helped us to dissociate the location, the hierarchical level, and the object on which the T1 and T2 letters materialized. Two grids masks were presented, on separate sides of an occlusion bar and with different colors (see Figure 8). A brief T1 appeared at one side, the grids then approached each other disappearing under the bar, later returning to their original position, where a brief T2 was revealed on one side. The colors of the grids were either kept constant (inducing a bounce percept) or swapped between objects (inducing a slide percept). T1 and T2 locations, level, and object/color were balanced across trials in a pseudo-random order. Thus, it was possible to study the effect of level, object, or location repetitions (and their interactions) on the attentional blink. If space plays a role in stimulus selection, changing target location should elicit an attentional blink even if the object and level are kept constant. If pure SF selection is present, then changing the level without changing location or object should elicit an attentional blink. Finally, if object-based selection plays a role, then changing the object (without changing level or location) should also trigger an attentional blink.

### Methods

#### Participants

Two groups of participants with ages between 25 and 36 years, were recruited with criteria equivalent to those of Experiment 1. One group viewed global T1s (11 subjects, 4 females). The other group viewed local T1s (12 subjects, 5 females). It was necessary to partition the design this way in order to avoid subject fatigue because of the large number of conditions involved. Overall, 14 of them also participated in other experiments reported in this article.

#### Stimuli

All stimuli were presented in the center of a CRT screen, placed 40 cm in front of the observers. Both groups of participants were tested with the same five global or local letters (and with the same sizes) used in Experiment 1 (E, H, S, P, U) and the grid mask.

#### Procedure

The trials began with a word (“left” or “right”) that cued the side of T1 presentation. Participants were asked to maintain fixation on the center of the screen and to press the spacebar on the computer keyboard to continue the trial. After this, two tokens of the grid mask were presented on a black background, one colored red and the other green. The inner border of each token abutted one side of a centrally placed white rectangle (140 mm high and 38 mm wide). Thus, the center of the tokens were shifted about 5 degrees from fixation. The lateralization of colors was randomly selected across trials. After 300 ms, one of the tokens was randomly selected and briefly transformed into a T1 (hierarchical level determined by group membership, durations by the titration described above). The mask baseline was then restored and both objects moved toward the center of the screen during 135 ms (at about 37°/s) until they were completely occluded by the central rectangle. This occlusion lasted 200 ms (a delay selected in a pilot study of 5 different subjects to maximize control of bounce/slide percepts). After this both tokens returned to their original position during the next 135 ms. In the bounce condition, they retained their original colors. In the slide condition, they switched colors. Note that this inward/outward symmetric motion was intended to discourage lateralized eye-movements. Afterwards, a T2 was then displayed at either the global or local level in one of the tokens that was randomly selected. After 300 ms of mask presentation, the trials were terminated when the participants had typed T1 and T2 identity on the computer keyboard.

The proportion of correct T2 identifications (only for trials with correct T1 responses) were submitted to an ANOVA using one between-subjects factor, T1-level (global vs. local), and three within-subjects factors: Object (same vs. different), Location (same vs. different), Level (same vs. different) and T2-side (left vs. right). The same ANOVA also was performed collapsing Group membership.

### Results and discussion

All subjects consistently perceived the slide and bounce percepts in the corresponding conditions. The main effect of Group (local T1 vs. global-T1) on T2 accuracy was not significant [*F*_(1, 21)_ = 1.72], and although some of its interactions with other factors were significant, their effect sizes were small. Though these interactions reflect difference in attentional blink size across trial types, this topic was examined more thoroughly in our previous experiments (in Experiment 1 with several T1–T2 SOAs, in contrast to only one here). Hence the repeated-measures ANOVA ignoring group/T1 level will be reported below, allowing us to concentrate of the interactions of Location, Level and Object.

**Figure 9:**
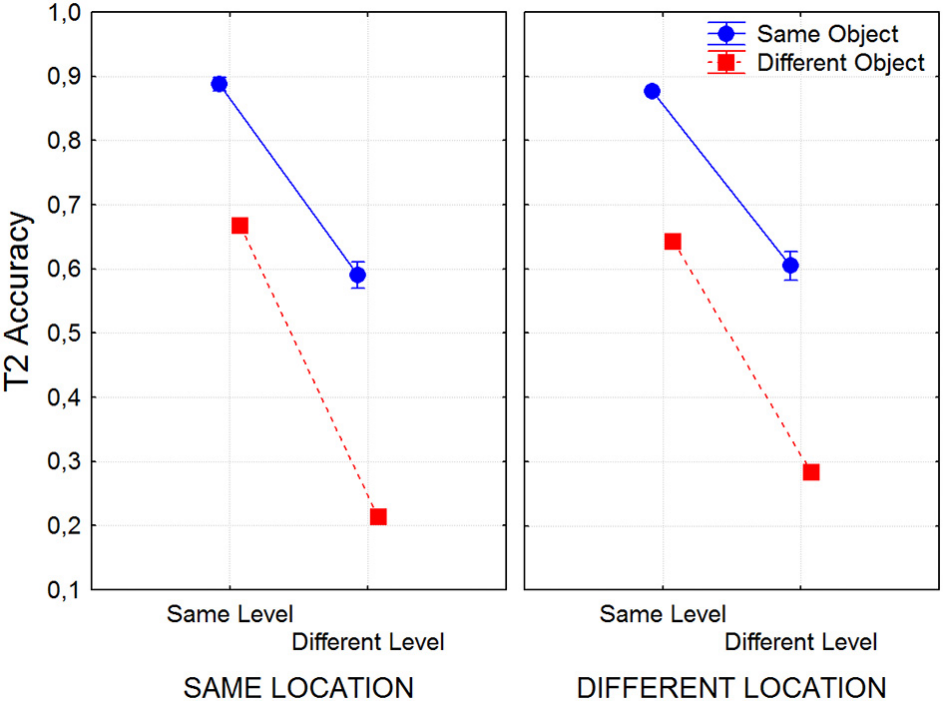
**T2 accuracy in Experiment 4 as a function of object, level and location repetition**.

The repetition of target level and target object both favored the accuracy of T2 reports (see Figure 9). The main effect of Level was highly significant [*F*_(1, 22)_ = 2260, *p* < 0.00001], with a large effect size (η^2^ = 0.55) replicating the same-level advantage found in the previous experiments reported above. Interestingly, the main effect of Object was also highly significant [*F*_(1, 22)_ = 827, *p* < 0.00001], with a large effect size (η^2^ = 0.38) due to more accurate T2 reports when the two targets affected the same object. Note that these effects of Object and Level were not simply additive. Their interaction was highly significant [*F*_(1, 22)_ = 9.28, *p* = 0.006], but of small effect size (η^2^ = 0.0006) and corresponds to a super-additive effect on the cost for T2 identification. Note, that the mean cost of changing only level was a drop of about 28% accuracy, that of changing only object was about 23%, but changing both level and object produced a drop of about 65%, almost sinking to chance level (20%). This interaction suggests that even if we choose to interpret the effect of level change as feature based mechanism (such as spatial frequency selection), it is not independent from the object selection. We suggest that here features contribute to attention as a consequence of their binding to an object-file.

The effect of Location was surprising. Instead of a cost, T2 reports were slightly favored by changing the placement of the targets. This effect of Location was significant [*F*_(1, 22)_ = 7.48, *p* < 0.0121], although with a small effect size (η^2^ = 0.0007). Moreover, the interactions of Location X Object [*F*_(1, 22)_ = 9.28, *p* < 0.00591, η^2^ = 0.0006], Location X Level [*F*_(1, 22)_ = 62.4, *p* = 0.00001, η^2^ = 0.0041], and Location X Level X Object [*F*_(1, 22)_ = 12.8, *p* = 0.00167, η^2^ = 0.0013] were all significant. Although all the effect sizes in these interactions were small, their nature was consistent. Shifting the location of the targets ameliorated the attentional blink elicited by a reduction of target similarity. This is best seen in Figure 9. Planned comparisons showed that the effect of Location on T2 reports was not significant for same-objects/same-levels, or for same-objects/different levels, but it was significant for different-objects/same-levels [*F*_(1, 21)_ = 13.03, *p* < 0.002], and highly significant for different-objects/different-levels [*F*_(1, 21)_ = 59.69, *p* < 0.00001]. This last effect corresponded to a saving of about 8% in T2 recognition accuracy. T2 side was also significant [*F*_(1, 22)_ = 12.7, *p* = 0.00173, η^2^ = 0.0007] due to a very small advantage (about 1%) for targets placed on the left side. Interactions of T1 side with Location, Object, Location X Level were also significant but with very small effect sizes.

This experiment again elicited a large attentional blink when hierarchal level changed from T1 to T2. This effect was present even when the location and the object did not change. This is consistent with feature-based selection. A large attentional blink was also generated when the targets switched objects, and the effect was present even when level and location were invariant. This is consistent with object-based attention, and to our knowledge the first evidence of its involvement in the perception of compound letters. Thus, pure feature selection cannot explain all the data. However, a switch in location did not generate an attentional blink *per se*. Thus, a classical “attentional spotlight” (Posner, 1980), cannot explain our results. In fact, location repetition produced a cost for T2 accuracy, an outcome opposite of what expected from a spotlight.

One could argue that what we call a shift in objects is really a color change (another feature). The subject could just be filtering out a color on each trial. This issue can be decisively resolved in future experiments if the two objects do not vary in any feature except their spatio-temporal trajectory (Pylyshyn and Storm, 1988; Kahneman et al., 1992). However, level and object (or color) repetition exhibit a super-additive interaction on T2 accuracy. Thus, this attentional blink must originate at a stage in both features (level and color) have been bound into an object-file. This idea of a bundle of features that survives displacements in space is precisely captured by the object-file concept. The results are consistent with the selection and a “sticky” engagement of attention with an individuated object. Perhaps SF is the medium by which object-files corresponding to different hierarchical levels are generated. According to this view the level effect observed in this experiment is simply due to the binding of specific SF channel to an object-file.

We have no clear explanation of the contra-intuitive finding that switching locations slightly ameliorates the massive attentional blink produced by a joint object/level switch. Perhaps the spatial-based-feature binding proposed by FIT (Treisman and Gelade, 1980) operates after object-file creation and leaves a location-bound trace. This would produce an additional cost on T2 recognition (that drops to chance level for a different-object/different-level, same-location as T1). This requires further research.

## Experiment 5

Task switching is seen as reconfiguration of executive processes (Monsell, 2003; Yeung and Monsell, 2003) due to endogenous control in anticipation of a given task. Although the basic task in our experiments (letter identification) is the same for both levels, the preparatory perceptual processes involved are radically different (Han et al., 1999; Han and Humphreys, 2002). For the local level the letters must be segmented, spatial reach scaled down, and information from irrelevant symbols filtered out. For the global letters the local elements must be integrated and irrelevant form details trimmed away (perhaps with a process akin to skeletonization, Blum, 1973). This suggests that task switching could contribute to the different-level attentional blink. This is consistent with an explanation of same-level priming in the Navon task that was proposed by Lamb and Yund (1996). They argued that slower responses when level was not repeated on successive trials, is a consequence of the need to mobilize inactive neural processes. Moreover, is evidence that even conventional attentional blink under some circumstances could be partly due to task switching effects (Enns et al., 2001; Kawahara et al., 2003). To explore the role of task switching we repeated Experiment 2 with invalid cues about T1 level in some trials. If the attentional blink in our work is explainable by endogenous reconfiguration of executive processes, then invalid cues for T1 should decrease recognition accuracy for this target, but should reduce the duration of the attentional blink for T2 recognition on different level-trials (since for this type of target the cue would be valid).

### Methods

Five participants (3 females), with ages between 25 and 35, were recruited for this experiment. Two of them had also participated in other experiments reported in this article. The same procedures as in Experiment 2 were used, except for one modification. We manipulated the validity of the cue word (“GLOBAL” or “LOCAL”) that initiated each trial and which served to forewarn T1 hierarchical level. In 75% of the trials the cue was valid and in 25% it was invalid. Note that cues invalid for T1 were also invalid for T2 in same-level trials, but correctly cued T2 on different-level trials.

### Results and discussion

Cue Validity had a highly significant effect on T1 accuracy [*F*_(1, 4)_ = 236.22, *p* < 0.001], with better performance in valid trials (mean = 94%) than in invalid trials (mean = 84%). Neither Mask-type nor Transition-type presented significant effects on T1 recognition and none of the interaction effects was significant at a 0.01 threshold. Cue Validity also increased the ISI threshold for 75% correct T2 recognition [*F*_(1, 4)_ = 237.23, *p* < 0.001]. Thresholds for invalid trials were about 40 ms longer than for valid trials. Note that this difference is much smaller than the effect obtained when comparing local/global and global/local trials in the main Experiment 2 (an increase of about 200 ms). Mask-type was also significant [*F*_(1, 4)_ = 13.29, *p* = 0.022], with longer ISIs for trials including the noise mask, compared with those including the grid mask, as well as Transition-type [*F*_(1, 4)_ = 2049.7, *p* < 0.001], with much longer ISIs for different-level transitions. T2-level was not significant. The following interactions were significant: between Mask-type and Transition-type [*F*_(1, 4)_ = 56.90, *p* < 0.002], between Mask-type and T2-level [*F*_(1,4)_ = 163.87, *p* < 0.001], between Mask-type, Transition-type and T2-level [*F*_(1, 4)_ = 33.37, *p* < 0.005], and importantly between Cue-Validity and Transition-type [*F*_(1, 4)_ = 1188.8, *p* < 0.001]. However, (and contrary to the predictions) cue always increased the duration of the attentional blink [about 14.9 ms for same-level trials, *F*_(1, 4)_ = 27.0, *p* < 0.01 and about 75 ms for different-level trials, *F*_(1, 4)_ = 541.2, *p* < 0.00002]. Equivalent results were obtained for the SOA thresholds.

Cue validity had small but significant effect on T1 accuracy and the size of the attentional blink on same-level trials, suggesting that endogenous task preparation was indeed present. However, invalid T1 cues did not benefit the different level T2s (which were themselves validly cued). Instead a moderate increase in attentional blink duration was found for this type of trial, which was opposite to our predictions. This suggests that endogenous task preparation does not play an important role in generation the attentional blink. Nevertheless, automatic reconfiguration of executive processes (exogenously driven by T1) would be consistent with these results.

## General discussion

We measured the difficulty of dividing attention between two letters presented in rapid succession, each selectively unveiled at only one level of a compound figure. Identification of the two targets was accurate when both were presented at the same level within each trial. However, a large attentional blink affected the second target if it appeared on a different level from the first, which was abolished if the latter was ignored. The direct estimation of attentional shift duration with the staircase procedure indicates that they take about 50 ms for the same-level trials and from about 300 to 500 ms for different-level trials. Additionally, if the target switched between distinct objects, an attentional blink was also elicited. Object-change and level change exhibited a super- additive interaction on this attentional blink. The attentional blink was larger when the masks temporally enclosing the target letters had a greater overlap in spatial frequency content with the second target. This effect was also found for isolated targets free from competition, if the effects of inattention were mimicked by reducing stimulus contrast. The large attentional blink in different-level trails was robust over different psychophysical procedures, and low-level stimulus properties, thus confirming and extending previous work (Lopez et al., 2002; White et al., 2009; Valdes-Sosa et al., 2014).

Spatial-based attentional mechanisms do seem to not play a role in our findings. The difficulty in switching attention between levels was present in Experiments 1 and 2 despite the fact that targets were placed within the same location. Moreover, a switch in location in Experiment 4 did not generate an attentional blink. This is congruent with reports that location shifts do not affect level-specific priming (Robertson, 1996), nor do they diminish biasing of attention toward LSF or HSF gratings by previous selection of one level of a compound letter (Flevaris et al., 2011b). Attentional “zooming” (c.f. Stoffer, 1993) has been proposed as the basis for the selection of hierarchical levels (with wide attentional windows for global and narrow windows for local letters). The results from Experiment 2 argue against this spatial-based mechanism. Local targets were accurately reported despite abrupt and random changes in their locations. This precluded the focusing of narrow beams of attention at fixed locations, a potentially useful strategy for traditional Navon figures and in our Experiments 1 and 4. The accurate report of local letters in Experiment 2 would have required their capturing these narrow beams of attention at unrealistic speeds. Therefore “zooming in” is not a valid explanation for the selection of the local level. This is in line with a report that letter size by itself does not bias attention toward specific spatial frequencies (Flevaris et al., 2011b).

Previous research has proposed that attentional selection of hierarchical level is based on increasing the gain ofspecific spatial frequency channels (Robertson, 1996; Lamb et al., 1998, 1999; Flevaris et al., 2010, 2011a,b), an increase that can endure over time. Stimuli would thus pull sensory gain toward channels corresponding to their dominant SF content. The large attentional blinks for different-level trials found in Experiments 1, 2 and 4 could be explained by this proposal, if we assume that each level is mainly carried by a specific SF band. T2 recognition would benefit from an increased gain for its spatial frequency channel when it is on the same level as T1, and would consequently suffer when it is not. Also, the immunity of these attentional blinks to changes in target locations is consistent with the idea that this spatial frequency filter applies across spatial positions (Robertson, 1996; Flevaris et al., 2011b). If our attentional blink reflects the same mechanism as level-specific priming, then our data indicates that it operates at much shorter time lags than previously described (but see discussion of Experiment 2 for an alternative interpretation).

However, this feature-based model does not explain the object-based attentional blink found in Experiment 4, when targets switched objects (despite keeping the level invariant). Nor does it explain the interaction between level change and object change effects on attentional blink size in that experiment. Furthermore, the interaction of Mask-type and T2 level found in Experiments 1 and 2 cannot be easily explained by modulations of a spatial frequency filter, at least without additional elaboration. Therefore, the whole story cannot be reduced to an attentional weighting of spatial frequency channels that is agnostic about objects.

In the introduction we conjectured that two different letters at the same level could be interpreted as mutations of the same object-file, whereas letters from different levels could not. Previous work has suggested that updating an object-file requires less attentional resources that its creation (Valdes-Sosa et al., 2000; Valdes Sosa et al., 2003; Pinilla et al., 2001; Raymond, 2003; Kellie and Shapiro, 2004; Mitchell et al., 2004; Ciaramitaro et al., 2011), and that object-file continuity is not affected by masking or occlusion (Scholl, 2007). Based on these assumptions, we propose the following model to understand our findings: (1) In absence of attentional competition, T1 captures attention automatically and thus easily generates an object-file with a specific format that binds (among other properties) knowledge about letter type and hierarchical level; (2) At short SOAs between targets, T2 will be at disadvantage with T1 in the competition for attention; (3) This competitive disadvantage has little effect if T2 is from the same level as T1 since only an undemanding update of a compatible object-file is needed, hence no attentional blink is produced; (4) If T2 is from a different level than T1, it will trigger an attention greedy formation of a new object-file because the existing format is incompatible, which under the competitive disadvantage will entail a large attentional blink; and (5) If T1 is placed in one of two simultaneously existing objects, the other will suffer from competitive inhibition, thus placing any subsequent T2 it hosts at a disadvantage.

This object-based account explains the attentional blink found trials found in Experiments 1, 2, and 4 for different level but not same-level trials, as well as the invariance of these results to changes in target location. It is true that these findings are also predictable on the spatial frequency filtering view described above, although not exactly for the same reasons. On our view, it is object-file format incompatibility that determines this outcome. An object-file account is the only explanation for the attentional blink produced when the targets switched objects in Experiment 4. The latter result cannot be explained by the spatial frequency selection model. The object-file account can also easily explain the effect of mask-type on T2 recognition. This effect is only present in different-level trials. In Experiment 3 we showed that Mask-type effect could be reproduced by reducing the contrast of isolated letters. This is compatible with the idea that masks hamper the formation of new object-files (as suggested by Wutz and Melcher, 2013), which would be especially true under conditions of attentional disadvantage (in our case present for different-level T2s, but absent for same-level T2s or T1s).

Nevertheless, the two views are not incompatible. Spatial frequency could contribute to differentiate the format of object-files for different hierarchical levels. The features needed to represent the overall shape of an object are not the same as those used to represent an ensemble of smaller entities (Treisman, 2006). This is idea is congruent with the super-additive interaction of object- and level-change effects on the attentional blink in Experiment 4. This interaction suggests that the attentional blink is generated at a processing stage where different features have been bound together, including some delimiting object individuality and those signaling hierarchical level (i.e., spatial frequency). Furthermore, it is revealing that in Experiment 3, object-file formation at each level was hampered most by the visual mask with which it had the largest overlap in spatial frequency. This supports the idea that object-files at each level are assembled using different spatial frequency bands, which would contribute to their incompatibility.

The role of task switching should also be considered in explaining our results. The need to sequentially mobilize distinct perceptual processes for different levels could have induced a task-switch cost. This explanation was originally proposed to explain same-level priming by Lamb and Yund (1996), and is consistent with the idea that the attentional blink is a limitation in re-configuring an input filter (Di Lollo et al., 2005) In Experiment 5, invalid cueing of T1 level slightly impaired its recognition. Therefore, endogenous task preparation was evinced. But this invalid cueing did not decrease the attentional blink for the T2 in different-level trials (the level of which did correspond to the cue). However, the results do not exclude a major role for reconfiguration of executive processes that would allow tackling each level. This reconfiguration could be exogenously triggered by T1, an idea that fits our data. The attentional blink we see in our data could be due to a mixture of costs, including purely object-based attention (see Experiment 4) as well as task switching of the kind suggested by Lamb and Yund (1996). Different weights of the sub-processes in the mixture (associated with different experimental conditions) could explain why the attentional blink is of typical duration in some circumstances (around 500 ms), but seems to linger for seconds in others (as in our Experiment 1; see also Robertson, 1996; Lawson et al., 1998).

Hübner and Volberg (2005) proposed that abstract level and letter identity were bound at a relatively late stage of hierarchical perception. For simplicity we proposed that this information was bound within object-files. However, a theoretical distinction has been made between object-files, and their subsequent transformation and storage in short, or long-term visual memory as object-tokens (Treisman, 1992; Zimmer and Ecker, 2010). It is not clear if level and identity are bound into files, tokens, or both. Further research is needed to clarify the mechanisms of this binding, in which object-files will play either a direct or supportive role.

## Concluding remarks

Studies of object-based attention and of attention to hierarchical levels have been traditionally isolated from each other. The two-sequential target test we presented here allows us to examine them within the same empirical framework. By controlling the direction of attentional shifts between hierarchical levels we effectively disassembled the traditional Navon task. The methods used here are also relevant for research on the neural basis of these attentional processes. By selectively unveiling information from a single level within a compound figure, we can separate the presentation of global and local aspects and precisely control their timing. This is not possible with conventional Navon figures. Adequate timing of stimuli is critical for the successful application of event related potential and functional magnetic resonance methods (for an example of this approach see Valdes-Sosa et al., 2014).

Our data indicates that object-files play a role in the attentional selection of hierarchical levels. We have extended object-file theory in an attempt to explain how this happens. Our model explains the same data as previous theories, in addition to results that are incompatible with them. However, these different views need not be mutually exclusive, and probably can be integrated into a shared conceptual structure.

### Conflict of interest statement

The authors declare that the research was conducted in the absence of any commercial or financial relationships that could be construed as a potential conflict of interest.

## References

[B1] Arnell K. M. Howe A. E. Joanisse M. F. Klein R. M. (2006). Relationships between attentional blink magnitude, RSVP target accuracy, and performance on other cognitive tasks. Mem. Cogn. 34, 1472–1483. 10.3758/BF03195912 17263072

[B2] Bertenthal B. I. Banton T. Bradbury A. (1993). Directional bias in the perception of translating patterns. Perception 22, 193–193. 10.1068/p220193 8474844

[B3] Blum H. (1973). Biological shape and visual science. J. Theor. Biol. 38, 205–287. 10.1016/0022-5193(73)90175-6 4689997

[B4] Cameron E. L. Tai J. C. Carrasco M. (2002). Covert attention affects the psychometric function of contrast sensitivity. Vision Res. 42, 949–967. 10.1016/S0042-6989(02)00039-1 11934448

[B5] Carrasco M. Ling S. Read S. (2004). Attention alters appearance. Nat. Neurosci. 7, 308–313. 10.1038/nn1194 14966522 PMC3882082

[B6] Carrasco M. Penpeci-Talgar C. Eckstein M. (2000). Spatial covert attention increases contrast sensitivity across the CSF: support for signal enhancement. Vision Res. 40, 1203–1215. 10.1016/S0042-6989(00)00024-9 10788636 PMC3825514

[B7] Chakravarthi R. VanRullen R. (2011). Bullet trains and steam engines: exogenous attention zips but endogenous attention chugs along. J. Vis. 11:12. 10.1167/11.4.12 21508269

[B8] Chung S. T. Legge G. E. Tjan B. S. (2002). Spatial-frequency characteristics of letter identification in central and peripheral vision. Vision Res. 42, 2137–2152. 10.1016/S0042-6989(02)00092-5 12207975

[B9] Ciaramitaro V. M. Mitchell J. F. Stoner G. R. Reynolds J. H. Boynton G. M. (2011). Object-based attention to one of two superimposed surfaces alters responses in human early visual cortex. J. Neurophysiol. 105, 1258–1265. 10.1152/jn.00680.2010 21228306 PMC3074415

[B10] Crewther D. P. Lawson M. L. Crewther S. G. (2007). Global and local attention in the attentional blink. J. Vis. 7, 1–12. 10.1167/7.14.9 18217804

[B11] Desimone R. Duncan J. (1995). Neural mechanisms of selective visual attention. Annu. Rev. Neurosci. 18, 193–222. 10.1146/annurev.ne.18.030195.001205 7605061

[B12] Di Lollo V. Kawahara J. I. Ghorashi S. S. Enns J. T. (2005). The attentional blink: resource depletion or temporary loss of control? Psychol. Res. 69, 191–200. 10.1007/s00426-004-0173-x 15597184

[B13] Duncan J. (1984). Selective attention and the organization of visual information. J. Exp. Psychol. Gen. 113:501. 10.1037/0096-3445.113.4.501 6240521

[B14] Duncan J. Ward R. Shapiro K. (1994). Direct measurement of attentional dwell time in human vision. Nature 369, 313–315. 10.1038/369313a0 8183369

[B15] Dux P. E. Marois R. (2009). The attentional blink: a review of data and theory. Attent. Percept. Psychophys. 71, 1683–1700. 10.3758/APP.71.8.1683 19933555 PMC2915904

[B16] Egeth H. E. Yantis S. (1997). Visual attention: control, representation, and time course. Annu. Rev. Psychol. 48, 269–297. 10.1146/annurev.psych.48.1.269 9046562

[B17] Enns J. T. Visser T. A. Kawahara J. I. Di Lollo V. (2001). Visual masking and task switching in the attentional blink, in The Limits of Attention: Temporal Constraints in Human Information Processing, 65–81 10.1093/acprof:oso/9780198505150.003.0004

[B18] Feldman J. Tremoulet P. D. (2006). Individuation of visual objects over time. Cognition 99, 131–165. 10.1016/j.cognition.2004.12.008 16545625

[B19] Flevaris A. V. Bentin S. Robertson L. C. (2010). Local or global? Attentional selection of spatial frequencies binds shapes to hierarchical levels. Psychol. Sci. 21, 424–431. 10.1177/0956797609359909 20424080 PMC2861790

[B20] Flevaris A. V. Bentin S. Robertson L. C. (2011a). Attentional selection of relative SF mediates global versus local processing: evidence from EEG. J. Vis. 11:11. 10.1167/11.7.11 21670096 PMC3250221

[B21] Flevaris A. V. Bentin S. Robertson L. C. (2011b). Attention to hierarchical level influences attentional selection of spatial scale. J. Exp. Psychol. Hum. Percept. Perform. 37, 12. 10.1037/a0019251 20718576 PMC3035717

[B22] Flevaris A. V. Martínez A. Hillyard S. A. (2014). Attending to global versus local stimulus features modulates neural processing of low versus high spatial frequencies: an analysis with event-related brain potentials. Front. Psychol. 5:277. 10.3389/fpsyg.2014.00277 24782792 PMC3988377

[B23] Flombaum J. I. Scholl B. J. Pylyshyn Z. W. (2008). Attentional resources in visual tracking through occlusion: the high-beams effect. Cognition 107, 904–931. 10.1016/j.cognition.2007.12.015 18289519

[B24] Greenhouse S. W. Geisser S. (1959). On methods in the analysis of profile data. Psychometrika 24, 95–112 10.1007/BF02289823

[B25] Han S. Humphreys G. W. (2002). Segmentation and selection contribute to local processing in hierarchical analysis. Q. J. Exp. Psychol. A 55, 5–21. 10.1080/02724980143000127 11873855

[B26] Han S. Humphreys G. W. Chen L. (1999). Parallel and competitive processes in hierarchical analysis: perceptual grouping and encoding of closure. J. Exp. Psychol. Hum. Percept. Perform. 25:1411. 10.1037/0096-1523.25.5.1411 10531666

[B27] Hübner R. Volberg G. (2005). The integration of object levels and their content: a theory of global/local processing and related hemispheric differences. J. Exp. Psychol. Hum. Percept. Perform. 31:520. 10.1037/0096-1523.31.3.520 15982129

[B28] Kahneman D. Treisman A. (1984). Changing views of attention and automaticity. Var. Attent. 1, 29.

[B29] Kahneman D. Treisman A. Gibbs B. J. (1992). The reviewing of object files: object-specific integration of information. Cogn. Psychol. 24, 175–219. 10.1016/0010-0285(92)90007-O 1582172

[B30] Kawachi Y. Kawabe T. Gyoba J. (2011). Stream/bounce event perception reveals a temporal limit of motion correspondence based on surface feature over space and time. Iperception 2, 428. 10.1068/i0399 23145236 PMC3485788

[B31] Kawahara J. I. Zuvic S. M. Enns J. T. Di Lollo V. (2003). Task switching mediates the attentional blink even without backward masking. Percept. Psychophys. 65, 339–351. 10.3758/BF03194565 12785064

[B32] Kellie F. J. Shapiro K. L. (2004). Object file continuity predicts attentional blink magnitude. Percept. Psychophys. 66, 692–712. 10.3758/BF03194912 15311667

[B33] Khoe W. Mitchell J. F. Reynolds J. H. Hillyard S. A. (2005). Exogenous attentional selection of transparent superimposed surfaces modulates early event-related potentials. Vision Res. 45, 3004–3014. 10.1016/j.visres.2005.04.021 16153678

[B34] Khoe W. Mitchell J. F. Reynolds J. H. Hillyard S. A. (2008). ERP evidence that surface-based attention biases interocular competition during rivalry. J. Vis. 8, 18.1–18.11. 10.1167/8.3.18 18484824

[B35] Kim N. Ivry R. B. Robertson L. C. (1999). Sequential priming in hierarchically organized figures: effects of target level and target resolution. J. Exp. Psychol. Hum. Percept. Perform. 25:715. 10.1037/0096-1523.25.3.715 10385984

[B36] Kimchi R. (2014). The perception of hierarchical structure, in Oxford Handbook of Perceptual Organization, ed Wagemans J. (Oxford: Oxford University Press).

[B37] Kravitz D. J. Behrmann M. (2011). Space-, object-, and feature-based attention interact to organize visual scenes. Attention Percept. Psychophys. 73, 2434–2447. 10.3758/s13414-011-0201-z 22006523 PMC3897470

[B38] Lamb M. R. London B. Pond H. M. Whitt K. A. (1998). Automatic and controlled processes in the analysis of hierarchical structure. Psychol. Sci. 9, 14–19 10.1111/1467-9280.00003 10696615

[B39] Lamb M. R. Yund E. W. (1996). Spatial frequency and attention: effects of level-, target-, and location-repetition on the processing of global and local forms. Percept. Psychophys. 58, 363–373. 10.3758/BF03206812 8935897

[B40] Lamb M. R. Yund E. W. Pond H. M. (1999). Is attentional selection to different levels of hierarchical structure based on spatial frequency? J. Exp. Psychol. Gen. 128:88. 10.1037/0096-3445.128.1.88 10100393

[B41] Lawson M. L. Crewther D. P. Duke C. C. Henry L. Kiely P. M. West S. J.. (1998). Attentional blink in global versus local attentional modes. Aust. N.Z. J. Ophthalmol. 26, S88–S90. 10.1111/j.1442-9071.1998.tb01384.x 9685034

[B42] Lawson M. L. Crewther S. G. Junghans B. M. Crewther D. P. Kiely P. M. (2005). Changes in ocular accommodation when shifting between global and local attention. Clin. Exp. Optom. 88, 28–32. 10.1111/j.1444-0938.2005.tb06660.x 15658923

[B43] Lopez K. Torres R. Valdés-Sosa M. (2002). Medición directa del tiempo de tránsito atencional entre distintos niveles de figuras jerárquicas: observadores normales y autistas. Rev. CENIC Cienc. Biol. 33, 111–117.

[B44] Majaj N. J. Pelli D. G. Kurshan P. Palomares M. (2002). The role of spatial frequency channels in letter identification. Vision Res. 42, 1165–1184. 10.1016/S0042-6989(02)00045-7 11997055

[B45] Mannos J. Sakrison D. J. (1974). The effects of a visual fidelity criterion of the encoding of images. Inf. Theory 20, 525–536 10.1109/TIT.1974.1055250

[B46] Mitchell J. F. Stoner G. R. Fallah M. Reynolds J. H. (2003). Attentional selection of superimposed surfaces cannot be explained by modulation of the gain of color channels. Vision Res. 43, 1323–1328. 10.1016/S0042-6989(03)00123-8 12742102

[B47] Mitchell J. F. Stoner G. R. Reynolds J. H. (2004). Object-based attention determines dominance in binocular rivalry. Nature 429, 410–413. 10.1038/nature02584 15164062

[B48] Mitroff S. R. Scholl B. J. Wynn K. (2004). Divide and conquer how object files adapt when a persisting object splits into two. Psychol. Sci. 15, 420–425. 10.1111/j.0956-7976.2004.00695.x 15147497

[B49] Mitroff S. R. Scholl B. J. Wynn K. (2005a). The relationship between object files and conscious perception. Cognition 96, 67–92. 10.1016/j.cognition.2004.03.008 15833307

[B50] Mitroff S. R. Scholl B. J. Wynn K. (2005b). One plus one equals one: the effects of merging on object files, in Poster Presented at the Annual Meeting of the Psychonomic Society (Toronto, ON).

[B51] Monsell S. (2003). Task switching. Trends Cogn. Sci. 7, 134–140 10.1016/S1364-6613(03)00028-7 12639695

[B52] Moore C. M. Mordkoff J. T. Enns J. T. (2007). The path of least persistence: object status mediates visual updating. Vision Res. 47, 1624–1630. 10.1016/j.visres.2007.01.030 17451777

[B53] Navon D. (1977). Forest before trees: the precedence of global features in visual perception. Cogn. Psychol. 9, 353–383 10.1016/0010-0285(77)90012-3

[B54] Neisser U. Becklen R. (1975). Selective looking: attending to visually specified events. Cogn. Psychol. 7, 480–494 10.1016/0010-0285(75)90019-5

[B55] Peterson M. A. (2001). Object perception, in Blackwell Handbook of Perception, Chapter 6, ed Goldstein E. B. (Oxford, UK: Blackwell Publishers), 168–203.

[B56] Peterson M. A. Kim J. H. (2001). On what is bound in figures and grounds. Vis. Cogn. 8, 329–348 10.1080/13506280143000034

[B57] Pinilla T. Cobo A. Torres K. Valdes-Sosa M. (2001). Attentional shifts between surfaces: effects on detection and early brain potentials. Vision Res. 41, 1619–1630. 10.1016/S0042-6989(01)00039-6 11348645

[B58] Posner M. I. (1980). Orienting of attention. Q. J. Exp. Psychol. 32, 3–25. 10.1080/00335558008248231 7367577

[B59] Pylyshyn Z. W. Storm R. W. (1988). Tracking multiple independent targets: evidence for a parallel tracking mechanism. Spat. Vis. 3, 179–197. 10.1163/156856888X00122 3153671

[B60] Raymond J. E. (2003). New objects, not new features, trigger the attentional blink. Psychol. Sci. 14, 54–59. 10.1111/1467-9280.01418 12564754

[B61] Raymond J. E. Shapiro K. L. Arnell K. M. (1992). Temporary suppression of visual processing in an RSVP task: an attentional blink? J. Exp. Psychol. Hum. Percept. Perform. 18, 849–860. 10.1037/0096-1523.18.3.849 1500880

[B62] Reynolds J. H. Alborzian S. Stoner G. R. (2003). Exogenously cued attention triggers competitive selection of surfaces. Vision Res. 43, 59–66. 10.1016/S0042-6989(02)00403-0 12505605

[B63] Robertson L. C. (1996). Attentional persistence for features of hierarchical patterns. J. Exp. Psychol. Gen. 125:227. 10.1037/0096-3445.125.3.227 8751819

[B64] Scholl B. J. (2007). Object persistence in philosophy and psychology. Mind Lang. 22, 563–591 10.1111/j.1468-0017.2007.00321.x

[B65] Scholl B. J. Pylyshyn Z. W. Feldman J. (2001). What is a visual object? Evidence from target merging in multiple object tracking. Cognition 80, 159–177. 10.1016/S0010-0277(00)00157-8 11245843

[B66] Sekuler A. B. Sekuler R. (1999). Collisions between moving visual targets: what controls alternative ways of seeing an ambiguous display? Perception 28, 415–432. 10.1068/p2909 10664783

[B67] Serences J. T. Schwarzbach J. Courtney S. M. Golay X. Yantis S. (2004). Control of object-based attention in human cortex. Cereb. Cortex 14, 1346–1357. 10.1093/cercor/bhh095 15166105

[B69] Shulman G. L. Sullivan M. A. Gish K. Sakoda W. J. (1986). The role of spatial-frequency channels in the perception of local and global structure. Perception 15, 259–273. 10.1068/p150259 3797200

[B70] Shulman G. L. Wilson J. (1987). Spatial frequency and selective attention to local and global information. Perception 16, 89–101. 10.1068/p160089 3671045

[B71] Srivastava P. Kumar D. Srinivasan N. (2010). Time course of visual attention across perceptual levels and objects. Acta Psychol. 135, 335–342. 10.1016/j.actpsy.2010.09.001 20937502

[B72] Stoffer T. H. (1993). The time course of attentional zooming: a comparison of voluntary and involuntary allocation of attention to the levels of compound stimuli. Psychol. Res. 56, 14–25. 10.1007/BF00572129 8310101

[B73] Treisman A. (1992). Perceiving and re-perceiving objects. Am. Psychol. 47:862. 10.1037/0003-066X.47.7.862 1497217

[B74] Treisman A. (2006). How the deployment of attention determines what we see. Vis. Cogn. 14, 411–443. 10.1080/13506280500195250 17387378 PMC1832144

[B75] Treisman A. M. Gelade G. (1980). A feature-integration theory of attention. Cogn. Psychol. 12, 97–136. 10.1016/0010-0285(80)90005-5 7351125

[B76] Valdes Sosa M. Bobes M. A. Rodriguez V. Acosta Y. Perez A. Iglesias J. (2003). The influence of scene organization on attention: psychophysics and electrophysiology, in Attention and Performance XX, eds Duncan J. Kanwisher N. (Oxford: Oxford University Press), 321–344.

[B77] Valdes-Sosa M. Cobo A. Pinilla T. (2000). Attention to object files defined by transparent motion. J. Exp. Psychol. Hum. Percept. Perform. 26, 488–505. 10.1037/0096-1523.26.2.488 10811159

[B78] Valdes-Sosa M. Iglesias J. Torres R. Trujillo-Barreto N. (2014). Switching attention between the local and global levels in visual objects, in Cognitive Electrophysiology of Attention: Signals of the Mind, eds Mangun G. R. (Oxford: Elsevier), 165–177.

[B79] Vecera S. P. Behrmann M. McGoldrick J. (2000). Selective attention to the parts of an object. Psychon. Bull. Rev. 7, 301–308. 10.3758/BF03212985 10909137

[B80] Watson A. B. Pelli D. G. (1983). QUEST: a Bayesian adaptive psychometric method. Percept. Psychophys. 33, 113–120. 10.3758/BF03202828 6844102

[B81] White S. O'Reilly H. Frith U. (2009). Big heads, small details and autism. Neuropsychologia 47, 1274–1281. 10.1016/j.neuropsychologia.2009.01.012 19428391

[B82] Willenbockel V. Sadr J. Fiset D. Horne G. O. Gosselin F. Tanaka J. W. (2010). The SHINE toolbox for controlling low-level image properties. hist 1, 2. 10.3758/BRM.42.3.671 20805589

[B83] Wutz A. Melcher D. (2013). Temporal buffering and visual capacity: the time course of object formation underlies capacity limits in visual cognition. Attent. Percept. Psychophys. 75, 921–933. 10.3758/s13414-013-0454-9 23568083 PMC3691495

[B84] Yeung N. Monsell S. (2003). Switching between tasks of unequal familiarity: the role of stimulus-attribute and response-set selection. J. Exp. Psychol. Hum. Percept. Perform. 29:455. 10.1037/0096-1523.29.2.455 12760628

[B85] Zimmer H. D. Ecker U. K. (2010). Remembering perceptual features unequally bound in object and episodic tokens: neural mechanisms and their electrophysiological correlates. Neurosci. Biobehav. Rev. 34, 1066–1079. 10.1016/j.neubiorev.2010.01.014 20138910

