# Supplementary material for: Attentional selection of levels within hierarchically organized figures is mediated by object-files
Source: Front Integr Neurosci. 2014 Dec 16;8:91. doi: 10.3389/fnint.2014.00091 (PMC4267176; doi:10.3389/fnint.2014.00091)
Supplement: Supplementary file 6 [file DataSheet1.DOCX]

***Supplementary Material***

**“Attentional selection of levels within hierarchically organized figures is mediated by object-files“**

**Mitchell Valdés-Sosa, Jorge Iglesias-Fuster and Rosario Torres**

Cuban Center for Neuroscience

Correspondence:

Mitchell Valdés-Sosa

E-mail: mitchell@cneuro.edu.cu

Movie 1: Example of trial from Experiment 1 using the grid mask.

Movie 2: Example of trial from Experiment 1 using the noise mask.

Movie 3: Example of trial from Experiment 2 using the grid mask.

Movie 4: Example of trial from Experiment 2 using the noise mask.

Movie 5: Stimuli used in Experiment 3 (without color changes)
